# Labdane-Type Diterpenes, Galangalditerpenes A–C, with Melanogenesis Inhibitory Activity from the Fruit of *Alpinia galanga*

**DOI:** 10.3390/molecules22122279

**Published:** 2017-12-20

**Authors:** Yoshiaki Manse, Kiyofumi Ninomiya, Ryosuke Nishi, Yoshinori Hashimoto, Saowanee Chaipech, Osamu Muraoka, Toshio Morikawa

**Affiliations:** 1 Pharmaceutical Research and Technology Institute, Kindai University, 3-4-1 Kowakae, Higashi-osaka, Osaka 577-8502, Japan; yoshiaki4849sa735@gmail.com (Y.M.); ninomiya@phar.kindai.ac.jp (K.N.); kojirou922@gmail.com (R.N.); yoshinorihashimoto0608@gmail.com (Y.H.); chaipechann@hotmail.com (S.C.); muraoka@phar.kindai.ac.jp (O.M.); 2 Antiaging Center, Kindai University, 3-4-1 Kowakae, Higashi-osaka, Osaka 577-8502, Japan; 3 Faculty of Agro-Industry, Rajamangala University of Technology Srivijaya, Thungyai, Nakhon Si Thammarat 80240, Thailand

**Keywords:** *Alpinia galanga*, melanogenesis inhibitor, galangaldeterpene, labdane-type diterpene

## Abstract

In our continuing study of biologically active natural products from the fruit of *Alpinia galanga* (Zingiberaceae), we newly isolated three new labdane-type diterpenes, termed galangalditerpenes A–C (**1**–**3**), along with four known sesquiterpenes (**4**–**7**) and two diterpenes (**8** and **9**). The stereostructures of **1**–**3** were elucidated on the basis of their spectroscopic properties. The melanogenesis inhibitory activities in theophylline-stimulated murine B16 melanoma 4A5 cells of these isolates, including the new diterpenes (**1**–**3**, IC_50_ = 4.4, 8.6, and 4.6 μM, respectively), were found to be more than 6–87-fold higher than that of arbutin (174 μM), a commercially available positive control.

## 1. Introduction

Labdanes, belonging to the bicyclic diterpenoid group, have been found as secondary metabolites in the tissues of fungi, insects, and marine organisms, and in essential oils, resins, and tissues of higher plants. Among plant materials, the gymnosperms, as well as the Asteraceae, Lamiaceae, and Zingiberaceae families, are the most important sources of labdane-type diterpenoids [1,2]. Labdanes have been reported to have a broad spectrum of biological activities, including antimicrobial, antiviral, cytotoxic, radical scavenging, anti-hypertensive, hepatoprotective, and anti-inflammatory activities [1,2]. The Zingiberaceae plant *Alpinia galanga* Swartz, which is known as Greater Galangal in English and Kulanjan in Hindi, is widely cultivated in China, India, and Southeast Asian countries such as Thailand, Indonesia, and the Philippines [3]. The fruit of this plant has been used for the treatment of stomachache, dyspepsia, emesis, diarrhea, asthma, osteoarthritis, and rheumatoid arthritis in several traditional medicine systems such as Ayurveda and Siddha [3,4,5,6,7,8,9,10]. In the course of our studies on the chemical constituents of *A. galanga*, we have isolated several phenylpropanoid, neolignan, and sesquineolignan constituents from 80% aqueous acetone extracts of the rhizome [11,12] and the fruit parts of *A. galanga* [13]. We have also reported that several phenylpropanoids showed gastroprotective [11], antiallergic [14], anti-inflammatory [12,15,16], and melanogenesis inhibitory activities [13]. Further separation of the constituents in the extract from the fruit part allowed us to isolate three new labdane-type diterpenes, galangalditerpenes A (**1**), B (**2**), and C (**3**), along with four known sesquiterpenes (**4**–**7**) and two known diterpenes (**8** and **9**). Here, we describe the isolation and structural elucidation of **1**–**3** as well as their inhibitory effects on theophylline-stimulated melanogenesis in mouse murine B16 melanoma 4A5 cells.

## 2. Results and Discussion

### 2.1. Isolation

**Figure 1:**
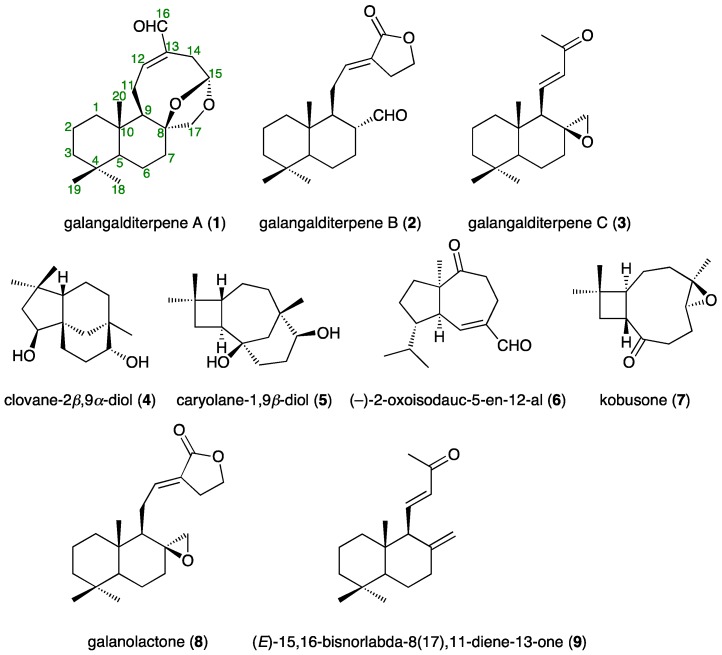
Structures of isolates (**1**–**9**) from the fruit of *A. galanga*.

In the present study, we isolated galangalditerpenes A (**1**, 0.00180%), B (**2**, 0.00121%), and C (**3**, 0.00475%), clovane-2β,9α-diol (**4**, 0.01341%) [17], caryolane-1,9β-diol (**5**, 0.00916%) [17], (−)-2-oxoisodauc-5-en-12-al (**6**, 0.00098%) [18], kobusone (**7**, 0.00066%) [17], galanolactone (**8**, 0.00148%) [7], and (*E*)-15,16-bisnorlabda-8(17),11-diene-13-one (**9**, 0.00136%) [19], from an 80% aqueous acetone extract of the fruit of *A. galanga* [13] using normal-phase silica gel and reversed-phase ODS CC, and finally preparative HPLC (Figure 1).

### 2.2. Structures of Galangalditerpenes A–C *(**1**–**3**)*

**Figure 2:**
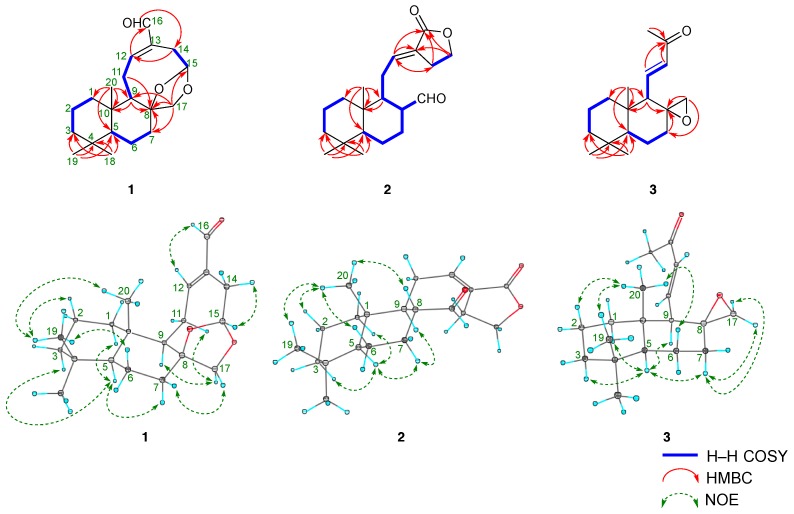
^1^H–^1^H COSY, HMBC, and NOESY correlations of **1**–**3**.

**Figure 3:**
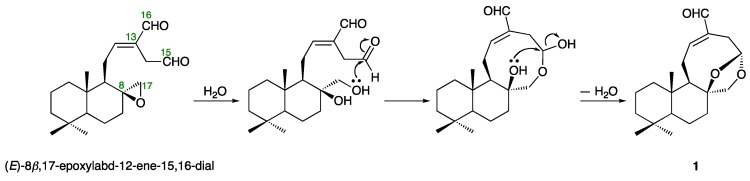
Hypothetical pathway to galangalditerpene A (**1**).

Galangalditerpene A (**1**) was obtained as a white powder with negative optical rotation ([α]${}_{D}^{25}$ −13.8 in CHCl_3_). The positive-ion EIMS spectrum of **1** showed a molecular ion peak at *m*/*z* 318 (M^+^), and the molecular formula was determined as C_20_H_30_O_3_ by high-resolution MS. The UV spectrum exhibited maximum absorption at 230 nm, while the IR spectrum showed absorption bands at 1686 cm^−1^ ascribable to the conjugated aldehyde function. The ^1^H- and ^13^C-NMR spectra of **1** (CDCl_3_, Table 1), which were assigned with the aid of distortion-less enhancement by polarization transfer (DEPT), ^1^H–^1^H correlation spectroscopy (COSY), heteronuclear multiple-quantum correlation (HMQC), and heteronuclear multiple bond correlation (HMBC) experiments (Figure 2 and Figure S1), showed signals assignable to three methyls (δ 0.80, 0.81, and 0.89 (3H each, all s, H_3_-18, 20, and 19)), six methylenes and a methylene bearing an oxygen function (δ (1.04 (1H, ddd, *J* = 3.2, 12.8, 12.8 Hz), 1.87 (1H, ddd, *J* = 2.4, 3.2, 12.8 Hz), H_2_-1), (1.15 (1H, dddd, *J* = 2.4, 3.2, 12.8, 13.6 Hz), 1.74 (1H, dddd, *J* = 2.4, 4.4, 4.4, 13.6 Hz), H_2_-6), (1.19 (1H, ddd, *J* = 4.0, 13.6, 13.6 Hz), 1.41 (ddd, *J* = 2.4, 2.4, 13.6 Hz), H_2_-3), (1.51 (1H, ddddd, *J* = 2.4, 3.2, 12.8, 13.6, 14.4 Hz), 1.60 (1H, m), H_2_-2), (1.64 (1H, m), 1.84 (1H, ddd, *J* = 2.4, 3.2, 12.8 Hz), H_2_-7), (2.27 (1H, ddd, *J* = 2.4, 3.2, 16.0 Hz), 3.13 (1H, dd, *J* = 5.6, 16.0 Hz), H_2_-14), (2.40 (1H, br dd, *J* = ca. 9, 13 Hz), 2.57 (1H, ddd, *J* = 8.8, 10.4, 12.8 Hz), H_2_-11), 3.62 and 4.35 (1H each, both d, *J* = 9.6 Hz, H_2_-17)), two methines and an acetal methine (δ 0.96 (1H, dd, *J* = 2.4, 12.8 Hz, H-5), 1.64 (1H, m, H-9), 5.44 (1H, dd, *J* = 3.2, 5.6 Hz, H-15)), a tri-substituted olefin (δ 6.87 (1H, ddd, *J* = 2.4, 8.8, 8.8 Hz, H-12)), and an aldehyde function (δ 9.35 (1H, s, H-16)). As shown in Figure 2 and Figure S1, the ^1^H–^1^H COSY experiment on **1** indicated the presence of partial structures, shown by bold lines. In the HMBC experiment on **1**, long-range correlations were observed between the following proton and carbon pairs: H-7 and C-8; H-9 and C-8, 10; H-12 and C-16; H-14 and C-12; H-16 and C-14; H_2_-17 and C-7–9, 15; H_3_-18 and C-3–5, 19; H_3_-19 and C-3–5, 18; H_3_-20 and C-1, 5, 9, 10. Thus, the linkage positions of the quaternary carbons (C-4, 8, 10, and 13) were clarified unambiguously, and the planar structure of **1** was elucidated. Next, the stereostructure of **1** was investigated using a nuclear Overhauser effect spectroscopy (NOESY) experiment, in which NOE correlations were observed between the following proton pairs: H-1α (δ 1.04) and H-5; H-2β (δ 1.51) and H_3_-19; H-3α (δ 1.19) and H-5; H-5 and H-7α (δ 1.64); H-6α (δ 1.15) and H_3_-19; H-7β (δ 1.84) and H-17β (δ 4.35); H-9 and H-17α (δ 3.62); H-12 and H-16; H-14α (δ 2.27) and H-15; H_3_-19 and H_3_-20, as shown in Figure 2. A hypothetical pathway from the corresponding diterpene (*E*)-8β,17-epoxylabd-15,16-dial, which was isolated from the seeds of *A. galanga* [7], to **1** is proposed in Figure 3. On the basis of the above-mentioned evidence, the stereostructure of **1** was determined to be as shown. To the best of our knowledge, this is the first report of the isolation of a labdane-type diterpene having a 5-formyl-8,10-dioxabicyclo[5.2.1]dec-4-en structure from natural sources.

**Scheme 1:**
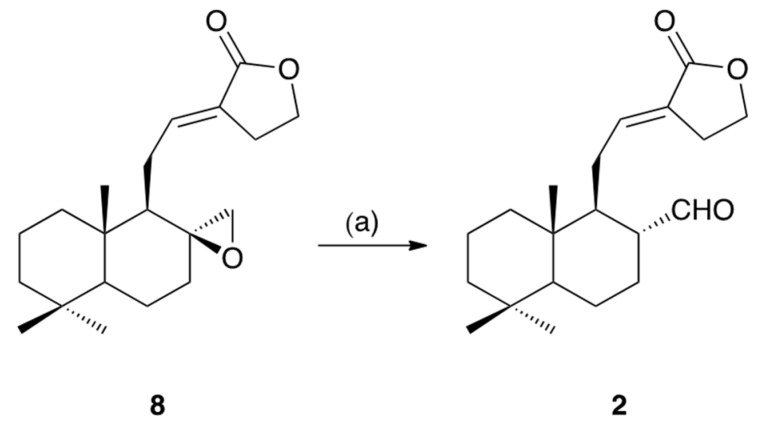
*Reagent and condition*: (a) BF_3_·Et_2_O/toluene, 0–10 °C, 30 min, 23%.

Galangalditerpene B (**2**) was obtained as a colorless oil with negative optical rotation ([*α*]${}_{D}^{25}$ −2.8 in CHCl_3_). In the positive-ion ESIMS profile, a sodium adduct ion peak was observed at *m*/*z* 341 [M + Na]^+^, and high-resolution ESIMS analysis revealed the molecular formula to be C_20_H_30_O_3_. The UV spectrum exhibited absorption maximum at 230 nm, while the IR spectrum showed absorption bands at 1728, 1712, and 1647 cm^−1^ ascribable to α,β-unsaturated lactone and aldehyde functions. The ^1^H- and ^13^C-NMR spectroscopic properties of **2** were generally superimposable onto those of galanolactone (**8**), but a difference was due to the 17α-formyl group in **2**. As shown in Table 1 (in CDCl_3_), these were three methyls (δ 0.84, 0.87, and 0.89 (3H each, all s, H_3_-18, 20, and 19)), seven methylenes and a methylene bearing an oxygen function (δ (1.02 (1H, ddd, *J* = 4.0, 12.8, 12.8 Hz), 1.76 (1H, m), H_2_-1), (1.20 (1H, ddd, *J* = 4.0, 13.4, 13.4 Hz), 1.76 (1H, ddd, *J* = 2.4, 3.6, 13.4 Hz), H_2_-3), (1.35 (1H, dddd, *J* = 4.0, 12.0, 12.4, 12.4 Hz), 1.74 (1H, m), H_2_-6), (1.40 (1H, dddd, *J* = 4.0, 12.0, 12.4, 12.4 Hz), 1.74 (1H, m), H_2_-7), (1.50 (1H, ddddd, *J* = 3.6, 4.0, 12.8, 13.4, 14.4 Hz), 1.76 (1H, ddddd, *J* = 2.4, 3.2, 4.0, 4.0, 14.4 Hz), H_2_-2), (1.81 (1H, ddd, *J* = 6.4, 9.6, 12.8 Hz), 2.40 (1H, ddd, *J* = 3.2, 6.4, 12.8 Hz), H_2_-11), 2.70 and 2.74 (1H each, both m, H_2_-14), (4.34 (1H, ddd, *J* = 6.4, 7.2, 14.4 Hz), 4.37 (1H, ddd, *J* = 6.4, 7.6, 14.4 Hz), H_2_-15)), three methines (δ 0.96 (1H, dd, *J* = 2.4, 12.4 Hz, H-5), 1.54 (1H, ddd, *J* = 3.2, 6.4, 14.4 Hz, H-9), 2.28 (1H, dddd, *J* = 3.2, 5.6, 12.0, 14.4 Hz, H-8)), a tri-substituted olefin (δ 6.62 (1H, dd, *J* = 6.4, 9.6 Hz, H-12)), and an aldehyde function (δ 9.28 (1H, d, *J* = 5.6 Hz, H-17)). As shown in Figure 2 and Figure S1, the connectivity of the quaternary carbons in **2** was determined using ^1^H–^1^H COSY and HMBC experiments. NOE correlations in the NOESY experiment were observed between the following proton pairs, as shown in Figure 2: H-1α (δ 1.02) and H-5; H-2β (δ 1.50) and H_3_-20; H-3α (δ 1.20) and H-5; H-5 and H-7α (δ 1.40); H-6β (δ 1.35) and H_3_-20; H-7α (δ 1.40) and H-9; H-8 and H_3_-20; H_3_-19 and H_3_-20, and on this basis, the relative stereostructure of **2** was determined. To elucidate the absolute stereostructure, we carried out the conversion of **8** into **2**. As shown in Scheme 1, the corresponding epoxide **8**, the absolute configuration of which has been reported [20], was rearranged in the presence of boron trifluoride diethyl ether complex (BF_3_·Et_2_O) to afford **2** [21]. Consequently, the absolute stereostructure of **2** was determined to be 5*S*, 8*R*, 9*S*, and 10*R*.

**Scheme 2:**
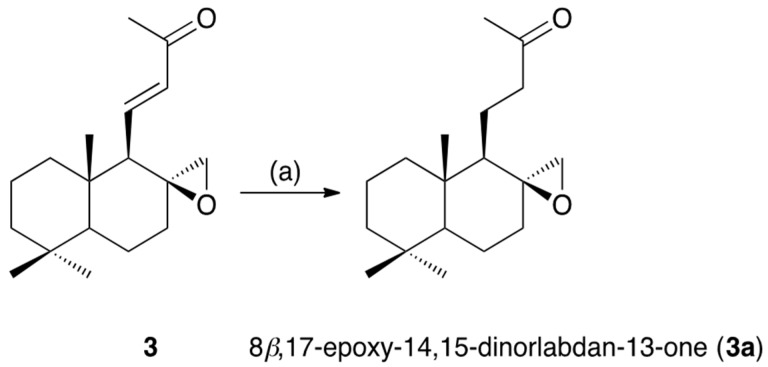
Reagent and condition: (a) H_2_, 5% Pd/C(en)/THF, r.t., 6 h, 90%.

Galangalditerpene C (**3**) was obtained as a colorless oil with positive optical rotation ([*α*]${}_{D}^{25}$ +6.1 in CHCl_3_), and its molecular formula, C_18_H_28_O_2_, was determined using EIMS and high-resolution EIMS measurements. The ^1^H- and ^13^C-NMR spectra of **3** (CDCl_3_, Table 1) showed signals assignable to four methyls (δ 0.90, 0.91, 1.09, 2.23 (3H each, all s, H_3_-18, 19, 20, and 16)), five methylenes and a methylene bearing an oxygen function (δ (0.96 (1H, ddd, *J* = 3.6, 12.8, 12.8 Hz), 1.42 (1H, m), H_2_-1), (1.17 (1H, ddd, *J* = 3.2, 13.6, 13.6 Hz), 1.43 (1H m), H_2_-3), 1.41 and 1.51 (1H each, both m, H_2_-2), (1.42 (1H, m), 1.97 (1H, ddd, *J* = 5.6, 13.6, 14.4 Hz), H_2_-7), (1.66 (1H, dddd, *J* = 4.0, 12.0, 13.6, 14.4 Hz), 1.71 (1H, br ddd, *J* = ca. 2, 6, 14 Hz), H_2_-6), and 2.31 and 2.38 (1H each, both d, *J* = 4.8 Hz, H_2_-17)), two methines (δ 1.00 (1H, dd, *J* = 2.4, 12.0 Hz, H-5), 1.42 (1H, br d, *J* = ca. 10 Hz, H-9)), and a di-substituted *trans*-olefin (δ 6.01 (1H, d, *J* = 16.8 Hz, H-12), 6.49 (1H, dd, *J* = 10.4, 16.8 Hz, H-11)). The ^1^H and ^13^C-NMR spectroscopic properties of **3** were quite similar to those of (*E*)-15,16-bisnorlabda-8(17),11-diene-13-one (**9**), except for the signal due to the 17-position [19]. As shown in Figure 2, the ^1^H–^1^H COSY experiment indicated the presence of partial structures, shown in bold lines. In the HMBC experiment, long-range correlations were observed as shown in Figure 2. Thus, the linkage positions of the quaternary carbons in **3** were determined unambiguously. The configuration of **3** was characterized using a NOESY experiment, which showed NOE correlations between the following proton pairs, as shown in Figure 2: H-1α (δ 0.96) and H-5; H-2β (δ 1.41) and H_3_-20; H-3α (δ 1.17) and H-5; H-5 and H-7α (δ 1.97), H-9; H-6β (δ 1.66) and H_3_-20; H-7α and H-17α (δ 2.31); H-7β (δ 1.42) and H-17α; H_3_-19 and H_3_-20. Finally, the absolute stereostructure of **3** was determined as chemically related to **3a**, the absolute configuration of which has been reported [22]. As shown in Scheme 2, the hydrogenation of **3** in the presence of 5% palladium-carbon (Pd/C)-ethylenediamine complex catalyst [Pd/C(en)] in tetrahydrofuran (THF) yielded **3a** [23]. Consequently, the absolute stereostructure of **3** was determined to be 5*S*, 8*S*, 9*R*, and 10*S*.

### 2.3. Effects on Theophylline-Stimulated Melanogenesis Inhibitory Activity

Melanin is a broad term for a group of natural pigments found in bacteria, fungi, plants, and animals. It is a heterogeneous, polyphenol-like biopolymer with a complex structure; the color varies from yellow to black through its development. The colors of mammalian skin and hair are determined by several factors, the most important being the degree and distribution of melanin pigmentation. The role of melanin is to protect the skin from ultra-violet (UV) damage by absorbing UV light and removing reactive oxygen species. However, excess production of melanin due to prolonged exposure to sunlight causes dermatological disorders such as melisma, freckles, post-inflammatory melanoderma, and solar lentigines. Melanin is secreted from melanocytes distributed in the basal layer of the dermis. Melanocytes are known to be stimulated by various factors, including UV radiation, α-melanocyte-stimulating hormone (α-MSH), or a phosphodiesterase inhibitor, theophylline [13,24,25,26,27,28,29]. In our previous investigation of naturally occurring compounds possessing melanogenesis inhibitory activity, we reported that several alkaloids [24,25,26,28], phenylethanoid glycosides [26], phenylpropanoids [13], neolignans [13], methoxyflavones [27], and diterpenes [29] exhibited significant positive effects against theophylline-stimulated melanogenesis in B16 melanoma 4A5 cells. As a continuation of the above study, melanogenesis inhibitors from the fruit of *A. galanga* were further explored. The results revealed that galangalditerpenes A (**1**, IC_50_ = 4.4 μM), B (**2**, 8.6 μM), and C (**3**, 4.6 μM), clovane-2β,9α-diol (**4**, 17.7 μM), caryolane-1,9β-diol (**5**, 9.4 μM), (−)-2-oxoisodauc-5-en-12-al (**6**, 2.9 μM), kobusone (**7**, 29.8 μM), galanolactone (**8**, 5.2 μM), and (*E*)-15,16-bisnorlabda-8(17),11-diene-13-one (**9**, 2.0 μM) showed potent activity without notable cytotoxic effects at effective concentrations (Table 2). The melanogenesis inhibitory activity was found to be more than 6–87-fold higher than that of arbutin (174 μM), a commercially available positive control [13,24,25,26,27,28,29].

### 2.4. Effects on Mushroom Tyrosinase

Tyrosinase, a copper-containing enzyme widely distributed in microorganisms, animals, and plants, is a key enzyme in melanin biosynthesis and determines the color of skin and hair. It catalyzes the oxidation of both l-tyrosine and l-DOPA, following the oxidation of l-DOPA to dopaquinone and oxidative polymerization via several dopaquinone derivatives to yield melanin. Tyrosinase inhibitors are clinically used for the treatment of several dermatologic disorders associated with melanin hyperpigmentation. In addition, these inhibitors are commonly used as additives in cosmetics for skin whitening and/or depigmentation [13,24,26,27,28,29]. As shown in Table S1, none of the isolates (**1**–**9**) showed inhibitory activity at effective concentrations when either l-tyrosine or l-DOPA was used as a substrate. This suggests that tyrosinase inhibition is minimally involved in the mechanisms of action of these melanogenesis inhibitors.

## 3. Materials and Methods

### 3.1. General Experimental Procedures

The following instruments were used to obtain physical data: specific rotation, JASCO P-2200 polarimeter (JASCO Corporation, Tokyo, Japan, *l* = 5 cm); UV spectra, Shimadzu UV-1600 spectrometer; IR spectra, IRAffinity-1 spectrophotometer (Shimadzu Corporation, Kyoto, Japan); EIMS and HREIMS, JMS-GC-MATE mass spectrometer (JEOL Ltd., Tokyo, Japan); ESIMS and HRESIMS, Exactive Plus mass spectrometer (Thermo Fisher Scientific Inc., Waltham, MA, USA); ^1^H-NMR spectra, JNM-ECA800 (800 MHz) and JNM-ECS400 (400 MHz) spectrometers (JEOL Ltd., Tokyo, Japan); ^13^C-NMR spectra, JNM-ECA800 (200 MHz) and JNM-ECS400 (100 MHz) spectrometers (JEOL Ltd., Tokyo, Japan) with tetramethylsilane as an internal standard; HPLC detector, SPD-10Avp UV-VIS detector (Shimadzu Co., Kyoto, Japan); HPLC column, Cosmosil 5C_18_-MS-II (Nacalai Tesque, Inc., Kyoto, Japan), 4.6 mm × 250 mm i.d. and 20 mm × 250 mm i.d. for analytical and preparative studies, respectively.

The following experimental conditions were used for chromatography (CC): ordinary-phase silica gel column chromatography, silica gel 60 N (Kanto Chemical Co., Tokyo, Japan; 63–210 mesh, spherical, neutral); reverse-phase silica gel CC, Chromatorex ODS DM1020T (Fuji Silysia Chemical, Aichi, Japan; 100–200 mesh); normal-phase TLC, pre-coated TLC plates with silica gel 60F_254_ (Merck, Darmstadt, Germany; 0.25 mm); reversed-phase TLC, pre-coated TLC plates with silica gel RP-18 F_254S_ (Merck, 0.25 mm); reversed-phase HPTLC, pre-coated TLC plates with silica gel RP-18 WF_254S_ (Merck, 0.25 mm); detection was carried out by spraying 1% Ce(SO_4_)_2_–10% aqueous H_2_SO_4_ onto the plates, followed by heating.

### 3.2. Plant Materials

The fruit of *Alpinia galanga* was collected in Nakhonsithammarat Province, Thailand, in September 2011. The plant material was identified by one of the authors (S.C.), and a voucher specimen (2011.09. Raj-01) of this plant is on file in our laboratory, as described in a previous report [13].

### 3.3. Extraction and Isolation

Dried fruit of *A. galanga* (3.0 kg) were extracted four times with 80% aqueous acetone at room temperature overnight. Evaporation of the combined extracts under reduced pressure provided an aqueous acetone extract (258.7 g, 8.62%). An aliquot (230.0 g) was partitioned into an EtOAc–H_2_O (1:1, *v*/*v*) mixture to furnish an EtOAc-soluble fraction (131.7 g, 5.09%) and an aqueous phase. The aqueous phase was subjected to Diaion HP-20 CC (3.0 kg, H_2_O → MeOH) to give H_2_O-eluted (19.4 g, 0.75%) and MeOH-eluted (24.8 g, 0.96%) fractions, respectively. An aliquot (110.0 g) of the EtOAc-soluble fraction was subjected to normal-phase silica gel CC [3.0 kg, n-hexane–EtOAc (10:1 → 5:1 →3:1 → 1:1, v/v) → EtOAc → MeOH] to give 11 fractions (Fr. 1 (1.16 g), Fr. 2 (2.91 g), Fr. 3 (2.95 g), Fr. 4 (6.45 g), Fr. 5 (9.80 g), Fr. 6 (11.47 g), Fr. 7 (1.25 g), Fr. 8 (10.03 g), Fr. 9 (2.80 g), Fr. 10 (16.28 g), and Fr. 11 (37.70 g)), as described previously [13]. Fraction 2 (2.91 g) was subjected to reversed-phase silica gel CC (90 g, MeOH–H_2_O (70:30 → 80:20 → 90:10, *v*/*v*) → MeOH) to afford eight fractions (Fr. 2-1 (113.1 mg), Fr. 2-2 (=(−)-2-oxoisodauc-5-en-12-al (**6**, 21.2 mg, 0.00098%)), Fr. 2-3 (35.7 mg), Fr. 2-4 (165.3 mg), Fr. 2-5 (77.5 mg), Fr. 2-6 (817.5 mg), Fr. 2-7 (823.7 mg), and Fr. 2-8 (850.0 mg)). Fraction 2-4 (165.3 mg) was purified by HPLC (MeOH–H_2_O (80:20, *v*/*v*)) to give galangalditerpene A (**1**, 4.0 mg, 0.00019%). Fraction 3 (2.95 g) was subjected to reversed-phase silica gel CC (90 g, MeOH–H_2_O (70:30 → 80:20 → 90:10, *v*/*v*) → MeOH) to afford six fractions (Fr. 3-1 (48.0 mg), Fr. 3-2 (94.1 mg), Fr. 3-3 (375.6 mg), Fr. 3-4 (274.9 mg), Fr. 3-5 (1244.7 mg), and Fr. 3-6 (860.0 mg)]. Fraction 3-3 (375.6 mg) was purified by HPLC (MeOH–H_2_O (80:20, *v*/*v*)) to give (*E*)-15,16-bisnorlabda-8(17),11-diene-13-one (**9**, 29.4 mg, 0.00136%). Fraction 4 (6.45 g) was subjected to reversed-phase silica gel CC (200 g, MeOH–H_2_O (60:40 → 80:20, *v*/*v*) → MeOH) to afford eight fractions (Fr. 4-1 (62.4 mg), Fr. 4-2 (2.06 g), Fr. 4-3 (239.1 mg), Fr. 4-4 (53.2 mg), Fr. 4-5 (802.6 mg), Fr. 4-6 (252.8 mg), Fr. 4-7 (187.6 mg), and Fr. 4-8 (2.80 g)), as described previously [13]. Fraction 4-5 (300.0 mg) was purified by HPLC (MeOH–H_2_O (75:25, *v*/*v*)] to give galangalditerpene C (**3**, 38.4 mg, 0.00475%). Fraction 4-6 (252.8 mg) was purified by HPLC (MeOH–H_2_O (80:20, *v*/*v*)) to give **1** (34.9 mg, 0.00161%). Fraction 5 (9.80 g) was subjected to reversed-phase silica gel CC (300 g, MeOH–H_2_O (60:40 → 80:20, *v*/*v*) → MeOH) to afford nine fractions (Fr. 5-1 (63.8 mg), Fr. 5-2 (323.8 mg), Fr. 5-3 (2.57 g), Fr. 5-4 (483.9 mg), Fr. 5-5 (223.9 mg), Fr. 5-6 (2.08 g), Fr. 5-7 (827.2 mg), Fr. 5-8 (140.8 mg), and Fr. 5-9 (2.78 g)), as described previously [13]. Fraction 5-4 (483.9 mg) was purified by HPLC (MeOH–H_2_O (60:40, *v*/*v*)) to give kobusone (**7**, 14.2 mg, 0.00066%). Fraction 5-6 (502.0 mg) was purified by HPLC (MeOH–H_2_O (80:20, *v*/*v*)) to give galangalditerpene B (**2**, 6.3 mg, 0.00121%) and galanolactone (**8**, 0.00148%), together with galanganol D diacetate (15.2 mg, 0.00292%) and *trans*-*p*-coumaryl acetate (0.00140%), as described previously [13]. Fraction 9 (2.80 g) was subjected to reversed-phase silica gel CC (90 g, MeOH–H_2_O (20:80 → 30:70 → 40:60 → 50:50 → 60:40 → 70:30, *v*/*v*) → MeOH) to afford seven fractions (Fr. 9-1 (419.0 mg), Fr. 9-2 (231.0 mg), Fr. 9-3 (= caryolane-1,9*β*-diol (**5**, 198.0 mg, 0.00916%)], Fr. 9-4 (20.8 mg), Fr. 9-5 ( = clovane-2*β*,9*α*-diol (**4**, 289.9 mg, 0.01341%)), Fr. 9-6 (658.7 mg), and Fr. 9-7 (947.1 mg)).

*Galangalditerpene A* (**1**): A white powder; [*α*]${}_{D}^{25}$ −13.8 (*c* 0.35, CHCl_3_); UV (MeOH, nm (log *ε*)): 230 (3.42); IR (KBr) *v*_max_ cm^−1^: 1686, 1649, 1136, 1103; ^1^H- and ^13^C-NMR spectroscopic data, see Table 1; EIMS *m*/*z* (%): 318 (M^+^, 3), 279 (9), 190 (100); HREIMS *m*/*z* 318.2197 (calcd. for C_20_H_30_O_3_, 318.2195).

*Galangalditerpene B* (**2**): A colorless oil; [*α*]${}_{D}^{25}$ −2.8 (*c* 0.29, CHCl_3_); UV (MeOH, nm (log *ε*)): 230 (3.40); IR (KBr) *v*_max_ cm^−1^: 1728, 1712, 1647; ^1^H- and ^13^C-NMR spectroscopic data, see Table 1; positive-ion ESIMS *m*/*z*: 341 [M + Na]^+^; HRESIMS *m*/*z*: 341.2104 [M + Na]^+^ (calcd. for C_20_H_30_O_3_Na, 341.2087).

*Galangalditerpene C* (**3**): A colorless oil; [*α*]${}_{D}^{25}$ +6.1 (*c* 0.47, CHCl_3_); UV (MeOH, nm (log *ε*)): 230 (3.71); IR (film) *v*_max_ cm^−1^: 1697, 1635, 1123; ^1^H- and ^13^C-NMR spectroscopic data, see Table 1; EIMS *m/z* (%): 276 (M^+^, 15), 258 (14), 43 (100); HREIMS *m*/*z* 276.2092 (calcd. for C_18_H_28_O_2_, 276.2089).

### 3.4. Conversion of Galanolactone *(**8**)* into Galangalditerpene B *(**2**)*

A solution of **8** (20.0mg, 0.06 mmol) in dry toluene (1.0 mL) at 0 °C was treated with BF_3_·Et_2_O (100 μL, 0.08 mmol), and the entire mixture was stirred at 0 °C for 30 min. The reaction mixture was poured into cold aqueous saturated NaHCO_3_ and extracted with EtOAc. The EtOAc extract was washed with brine, then dried over anhydrous Na_2_SO_4_ and filtered. Removal of the solvent under reduced pressure gave a crude product, which was purified by HPLC (CH_3_CN–H_2_O (55:45, *v*/*v*)) to furnish **2** (4.5 mg, 23%).

### 3.5. Hydrogenation of Galangalditerpene C *(**3**)*

A solution of **3** (5.5 mg, 0.02 mmol) in dry THF (1.0 mL) was treated with H_2_ in the presence of 5% Pd/C(en) (5.0 mg), and the entire mixture was stirred at room temperature for 6 h. The catalyst was removed by filtration, and the filtrate was evaporated under reduced pressure to give a crude product, which was purified by HPLC (MeOH–H_2_O (75:25, *v*/*v*)) to furnish **3a** (5.0 mg, 90%).

### 3.6. Reagents for Bioassays

Dulbecco’s modified Eagle’s medium (DMEM, 4.5 g/L glucose) was purchased from Sigma-Aldrich (St. Louis, MO, USA); fetal bovine serum (FBS), penicillin, and streptomycin were purchased from Gibco (Invitrogen, Carlsbad, CA, USA); and other chemicals used in this study were purchased from Wako Pure Chemical Co., Ltd. (Osaka, Japan). The 48- and 96-well microplates (Sumilon) were purchased from Sumitomo Bakelite Co., Ltd. (Tokyo, Japan).

### 3.7. Cell Culture

Murine B16 melanoma 4A5 cells (RCB0557) were obtained from Riken Cell Bank (Tsukuba, Japan). The cells were grown in DMEM (glucose; 4500 mg/L) supplemented with 10% FBS, penicillin (100 units/mL), and streptomycin (100 µg/mL) at 37 °C in 5% CO_2_/air. Cells were harvested by incubation in phosphate-buffered saline (PBS) containing 0.05% ethylenediaminetetraacetic acid (EDTA) and 0.02% trypsin for ca. 5 min at 37 °C and used for the subsequent bioassays.

### 3.8. Melanogenesis and Cell Viability

Effects on theophylline-stimulated melanogenesis and the viability of B16 melanoma 4A5 cells were determined as described previously [13,25,26,27,28,29].

### 3.9. Mushroom Tyrosinase

Tyrosinase activities using l-tyrosine or 3,4-dihydroxyphenyl-l-alanine (l-DOPA) as a substrate were determined as described previously [13,25,26,27,28,29].

### 3.10. Statistical Analysis

Values are expressed as mean ± S.E.M. One-way analysis of variance followed by Dunnett’s test was used for statistical analyses. Probability (*p*) values less than 0.05 were considered significant.

## 4. Conclusions

In conclusion, three new labdane-type diterpenes, galangalditerpenes A–C (**1**–**3**), were newly isolated from the 80% aqueous acetone extract of the fruit of *A. galanga* (Zingiberaceae) together with four known sesquiterpenes, clovane-2β,9α-diol (**4**), caryolane-1,9β-diol (**5**), (−)-2-oxoisodauc-5-en-12-al (**6**), and kobusone (**7**), and two diterpenes, galanolactone (**8**) and (*E*)-15,16-bisnorlabda-8(17),11-dien-13-one (**9**). The melanogenesis inhibitory activities in theophylline-stimulated murine B16 melanoma 4A5 cells of these isolates including the new diterpenes (**1**–**3**, IC_50_ = 4.4, 8.6, and 4.6 μM, respectively) were found to be more than 6–87-fold higher than that of arbutin (174 μM), a commercially used tyrosinase inhibitor. Considering the mechanisms of action, we examined the activity of the isolates against tyrosinase using either L-tyrosine or L-DOPA as a substrate. However, none of the isolates (**1**–**9**) showed inhibitory activity at the effective concentration. The detailed mechanisms of action of the melanogenesis inhibitory activity of the isolates require further examination.

## Figures and Tables

**Table 1:** NMR data (800 MHz, CDCl_3_) for galangalditerpenes A–C (**1**–**3**).

Position	1		2		3	
	δ_H_ (*J* in Hz)	δ_C_	δ_H_ (*J* in Hz)	δ_C_	δ_H_ (*J* in Hz)	δ_C_
1	1.04 (ddd, 3.2, 12.8, 12.8, Hα), 1.87 (ddd, 2.4, 3.2, 12.8, Hβ)	39.4	1.02 (ddd, 4.0, 12.8, 12.8, Hα), 1.76 (m, Hβ)	39.0	0.96 (ddd, 3.6, 12.8, 12.8, Hα), 1.42 (m, Hβ)	40.5
2	1.60 (m, Hα), 1.51 (ddddd, 2.4, 3.2, 12.8, 13.6, 14.4, Hβ)	18.6	1.76 (ddddd, 2.4, 3.2, 4.0, 4.0, 14.4, Hα), 1.50 (ddddd, 3.6, 4.0, 12.8, 13.4, 14.4, Hβ)	18.5	1.51 (m, Hα), 1.41 (m, Hβ)	18.3
3	1.19 (ddd, 4.0, 13.6, 13.6, Hα), 1.41 (ddd, 2.4, 2.4, 13.6, Hβ)	41.6	1.20 (ddd, 4.0, 13.4, 13.4, Hα), 1.76 (ddd, 2.4, 3.6, 13.4, Hβ)	41.8	1.17 (ddd, 3.2, 13.6, 13.6, Hα), 1.43 (m, Hβ)	41.9
4		33.4		33.3		33.5
5	0.96 (dd, 2.4, 12.8)	55.5	0.96 (dd, 2.4, 12.4)	54.5	1.00 (dd, 2.4, 12.0)	54.2
6	1.74 (dddd, 2.4, 4.4, 4.4, 13.6, Hα), 1.15 (dddd, 2.4, 3.2, 12.8, 13.6, Hβ)	20.5	1.74 (m, Hα), 1.35 (dddd, 4.0, 12.0, 12.4, 12.4, Hβ)	20.0	1.71 (br ddd, ca. 2, 6, 14, Hα), 1.66 (dddd, 4.0, 12.0, 13.6, 14.4, Hβ)	19.9
7	1.64 (m, Hα), 1.84 (ddd, 2.4, 3.2, 12.8, Hβ)	40.1	1.40 (dddd, 4.0, 12.0, 12.4, 12.4, Hα), 1.74 (m, Hβ)	26.8	1.97 (ddd, 5.6, 13.6, 14.4, Hα), 1.42 (m, Hβ)	18.3
8		84.7	2.28 (dddd, 3.2, 5.6, 12.0, 14.4)	53.8		58.2
9	1.64 (m)	57.4	1.54 (ddd, 3.2, 6.4, 14.4)	50.3	1.42 (br d, ca. 10)	56.9
10		39.5		37.5		39.5
11	2.40 (br dd, ca. 9, 13, Hα), 2.57 (ddd, 8.8, 10.4, 12.8, Hβ)	24.7	1.81 (ddd, 6.4, 9.6, 12.8), 2.40 (ddd, 3.2, 6.4, 12.8)	29.9	6.49 (dd, 10.4, 16.8)	144.4
12	6.87 (ddd, 2.4, 8.8, 8.8)	157.6	6.62 (dd, 6.4, 9.6)	139.6	6.01 (d, 16.8)	135.8
13		140.4		126.2		198.4
14	2.27 (ddd, 2.4, 3.2, 16.0, Hα), 3.13 (dd, 5.6, 16.0, Hβ)	29.6	2.70 (m), 2.74 (m)	25.3		
15	5.44 (dd, 3.2, 5.6)	100.4	4.34 (ddd, 6.4, 7.2, 14.4), 4.37 (ddd, 6.4, 7.6, 14.4)	65.4		
16	9.35 (s)	194.1		171.1	2.23 (3H, s)	26.4
17	3.62 (d, 9.6, Hα), 4.35 (d, 9.6, Hβ)	70.4	9.28 (d, 5.6)	203.8	2.31 (d, 4.8, Hα), 2.38 (d, 4.8, Hβ)	48.8
18	0.80 (3H, s)	21.5	0.84 (3H, s)	21.7	0.90 (3H, s)	21.9
19	0.89 (3H, s)	33.5	0.89 (3H, s)	33.4	0.91 (3H, s)	33.5
20	0.81 (3H, s)	14.9	0.87 (3H, s)	14.2	1.09 (3H, s)	15.6

**Table 2:** Inhibitory effects of the isolates (**1**–**9**) from the fruit of *A. galanga* on theophylline-stimulated melanogenesis and viability in B16 4A5 cells.

Treatment	Inhibition (%)					IC_50_ (µM)
	0 µM	3 µM	10 µM	30 µM	100 µM	
Galangalditerpene A (**1**)	0.0 ± 4.6 (100.0 ± 1.3)	48.3 ± 4.1 ** (95.1 ± 0.9)	56.6 ± 6.9 ** (91.4 ± 1.9)	67.9 ± 5.9 ** (78.4 ± 2.3 ^#^)	86.2 ± 4.4 ** (48.6 ± 1.1 ^#^)	4.4
Galangalditerpene B (**2**)	0.0 ± 9.3 (100.0 ± 3.9)	30.5 ± 9.1 ** (94.2 ± 6.1)	50.9 ± 3.4 ** (107.3 ± 3.9)	80.6 ± 2.3 ** (137.1 ± 1.6)	85.3 ± 2.6 ** (126.5 ± 1.7)	8.6
Galangalditerpene C (**3**)	0.0 ± 9.5 (100.0 ± 6.0)	40.6 ± 2.8 ** (94.2 ± 3.7)	67.1 ± 2.2 ** (114.9 ± 1.8)	82.5 ± 4.4 ** (126.9 ± 4.1)	— (13.1 ± 0.6 ^#^)	4.6
Clovane-2*β*,9*α*-diol (**4**)	0.0 ± 3.4 (100.0 ± 4.7)	23.9 ± 3.9 ** (98.1 ± 3.0)	37.2 ± 2.6 ** (93.8 ± 1.4)	64.0 ± 4.7 ** (102.3 ± 3.2)	73.6 ± 3.7 ** (76.2 ± 3.6 ^#^)	17.7
Caryolane-1,9*β*-diol (**5**)	0.0 ± 6.0 (100.0 ± 4.2)	37.3 ± 3.2 ** (98.4 ± 3.3)	53.8 ± 2.0 ** (98.2 ± 2.5)	52.7 ± 7.3 ** (105.3 ± 1.5)	87.9 ± 3.5 ** (96.7 ± 1.1)	9.4
(−)-2-Oxoisodauc-5-en-12-al (**6**)	0.0 ± 11.8 (100.0 ± 4.2)	45.9 ± 7.8 ** (87.7 ± 2.4)	67.8 ± 3.3 ** (86.3 ± 2.6)	85.1 ± 2.2 ** (90.0 ± 0.8)	86.7 ± 4.0 ** (59.9 ± 0.3 ^#^)	2.9
Kobusone (**7**)	0.0 ± 6.6 (100.0 ± 1.8)	−16.1 ± 3.6 (97.0 ± 2.1)	20.5 ± 8.3 * (97.3 ± 2.2)	51.5 ± 7.5 ** (92.1 ± 1.4)	82.8 ± 2.7 ** (87.5 ± 0.5)	29.8
Galanolactone (**8**)	0.0 ± 9.4 (100.0 ± 5.6)	38.6 ± 4.1 ** (95.3 ± 5.2)	64.4 ± 7.5 ** (113.6 ± 4.1)	83.8 ± 3.0 ** (134.4 ± 4.1)	— (39.9 ± 3.6 ^#^)	5.2
(*E*)-15,16-Bisnorlabda-8(17), 11-diene-13-one (**9**)	0.0 ± 5.8 (100.0 ± 2.3)	56.8 ± 3.4 ** (91.4 ± 1.3)	60.8 ± 2.2 ** (98.5 ± 1.6)	71.1 ± 1.4 ** (107.3 ± 2.0)	— (36.4 ± 1.6 ^#^)	2.0
Arbutin [13,24,25,26,27,28,29]	0.0 ± 1.4 (100.0 ± 2.1)	20.4 ± 0.5 (82.4 ± 3.0)	38.1 ± 0.9 ** (78.1 ± 1.9)	61.5 ± 0.6 ** (79.8 ± 2.2)	83.7 ± 0.5 ** (53.1 ± 1.8 ^#^)	174

Each value represents the mean ± S.E.M. (*n* = 4); asterisks denote significant differences from the control group, * *p* < 0.05, ** *p* < 0.01; ^#^ cytotoxic effects were observed, and values in parentheses indicate cell viability (%) in MTT assay; commercial arubutin was purchased from Nakalai Tesque Inc., (Kyoto, Japan).

## Supplementary Materials

Supplementary materials are available online. Figure S1: NMR spectra of galangalditerpenes A–C (**1**–**3**); Table S1: Effects of the isolates (**1**–**9**) from the fruit of *A. galanga* on the activity of tyrosinase from mushroom.
