# Supplementary material for: Labdane-Type Diterpenes, Galangalditerpenes A–C, with Melanogenesis Inhibitory Activity from the Fruit of Alpinia galanga
Source: Molecules. 2017 Dec 20;22(12):2279. doi: 10.3390/molecules22122279 (PMC6149739; doi:10.3390/molecules22122279)
Supplement: Supplementary file 1 [file molecules-22-02279-s001.zip › supplementary.v1.pdf]

Supplementary Materials:

Labdane-type Diterpenes, Galangalditerpenes A–C, with Melanogenesis Inhibitory Activity from the Fruit of *Alpinia galanga*

Yoshiaki Manse [1], Kiyofumi Ninomiya [1,2], Ryosuke Nishi [1], Yoshinori Hashimoto [1], Saowanee Chaipech [1,3], Osamu Muraoka [1,2] and Toshio Morikawa [1,2,*]

[1] Pharmaceutical Research and Technology Institute, Kindai University, 3-4-1 Kowakae, Higashi-osaka, Osaka 577-8502, Japan; yoshiaki4849sa735@gmail.com (Y.M.); ninomiya@phar.kindai.ac.jp (K.N.); kojirou922@gmail.com (R.N.); yoshinorihashimoto0608@gmail.com (Y.H.); chaipechann@hotmail.com (S.C.); muraoka@phar.kindai.ac.jp (O.M.)

[2] Antiaging Center, Kindai University, 3-4-1 Kowakae, Higashi-osaka, Osaka 577-8502, Japan

[3] Faculty of Agro-Industry, Rajamangala University of Technology Srivijaya, Thungyai, Nakhon Si Thammarat 80240, Thailand

* Correspondence: morikawa@kindai.ac.jp; Tel.: +81-6-4307-4306

Figure S1. NMR spectra of galangalditerpenes A–C (**1**–**3**)

Galangalditerpene A (**1**) · · · · · · · · · · · · · 1 – 6
- ^{1}H NMR spectrum · · · · · · · · · · · · · 1
- ^{13}C NMR and DEPT spectra · · · · · · · · · · · · · 2
- ^{1}H–^{1}H COSY spectrum · · · · · · · · · · · · · 3
- HSQC spectrum · · · · · · · · · · · · · 4
- HMBC spectrum · · · · · · · · · · · · · 5
- NOESY spectrum · · · · · · · · · · · · · 6

Galangalditerpene B (**2**) · · · · · · · · · · · · · 7 – 12
- ^{1}H NMR spectrum · · · · · · · · · · · · · 7
- ^{13}C NMR and DEPT spectra · · · · · · · · · · · · · 8
- ^{1}H–^{1}H COSY spectrum · · · · · · · · · · · · · 9
- HSQC spectrum · · · · · · · · · · · · · 10
- HMBC spectrum · · · · · · · · · · · · · 11
- NOESY spectrum · · · · · · · · · · · · · 12

Galangalditerpene C (**3**) · · · · · · · · · · · · · 13 – 18
- ^{1}H NMR spectrum · · · · · · · · · · · · · 13
- ^{13}C NMR and DEPT spectra · · · · · · · · · · · · · 14
- ^{1}H–^{1}H COSY spectrum · · · · · · · · · · · · · 15
- HSQC spectrum · · · · · · · · · · · · · 16
- HMBC spectrum · · · · · · · · · · · · · 17
- NOESY spectrum · · · · · · · · · · · · · 18

galangalditerpene A (1)

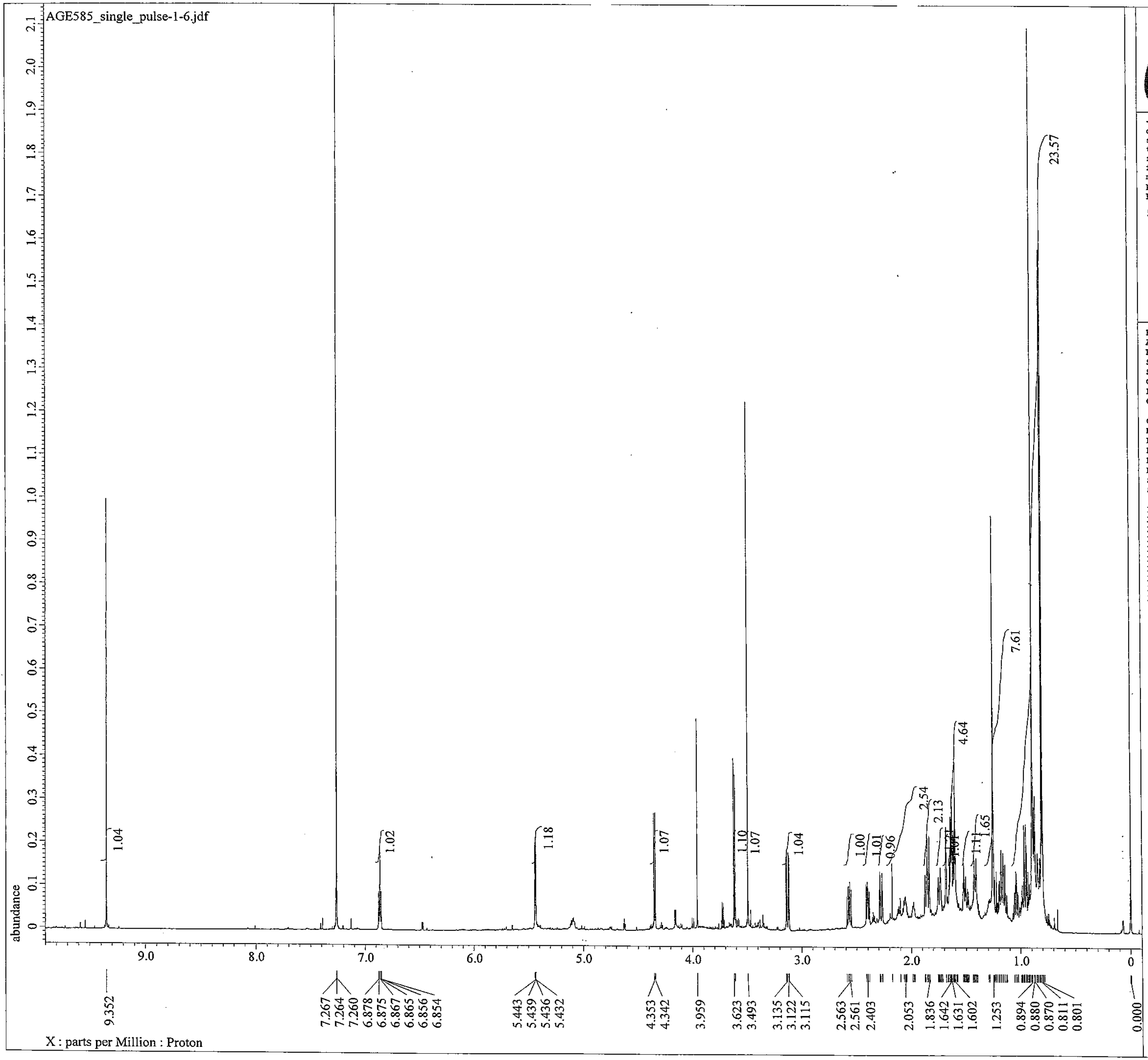


```
---- PROCESSING PARAMETERS ----
dc_balance( 0, FALSE )
sexp( 0.2[Hz], 0.0[s] )
trapezoid( 0[%], 0[%], 80[%], 100[%] )
zerofill( 2 )
fft( 1, TRUE, TRUE )
machinephase
ppm
phase( -3, 0, 35.02327[%] )
以下に由来:: AGE585_single_pulse-1-1.jdf
```

```
Filename          = C:\Users\datum\Documents\J
Author            = delta
Experiment        = single_pulse.jxp
Sample_Id         = AGE585
Solvent           = CHLOROFORM-D
Creation_Time     = 15-FEB-2012 12:47:44
Revision_Time     = 15-FEB-2012 13:00:54
Current_Time      = 15-FEB-2012 13:01:32

Comment           = single_pulse
Data_Format       = 1D COMPLEX
Dim_Size          = 52429
Dim_Title         = Proton
Dim_Units         = [ppm]
Dimensions        = X
Site              = JNM-ECA800
Spectrometer      = DELTA2_NMR

Field_Strength    = 18.79305132[T] (800[MHz])
X_Acq_Duration    = 2.18103808[s]
X_Domain          = 1H
X_Freq            = 800.14000039[MHz]
X_Offset          = 5[ppm]
X_Points          = 32768
X_Prescans        = 1
X_Resolution      = 0.45849727[Hz]
X_Sweep           = 15.02403846[kHz]
X_Sweep_Clipped   = 12.01923077[kHz]
Irr_Domain        = Proton
Irr_Freq          = 800.14000039[MHz]
Irr_Offset        = 5[ppm]
Tri_Domain        = Proton
Tri_Freq          = 800.14000039[MHz]
Tri_Offset        = 5[ppm]
Clipped           = FALSE
Scans             = 8
Total_Scans       = 8

Relaxation_Delay  = 5[s]
Recvr_Gain        = 48
Temp_Get          = 19.7[dC]
X_90_Width        = 14[us]
X_Acq_Time        = 2.18103808[s]
X_Angle           = 45[deg]
X_Atn             = 1.3[dB]
X_Pulse           = 7[us]
Irr_Mode          = Off
Tri_Mode          = Off
Dante_Presat      = FALSE
Initial_Wait      = 1[s]
Repetition_Time   = 7.18103808[s]
```

galangalditerpene A (1)

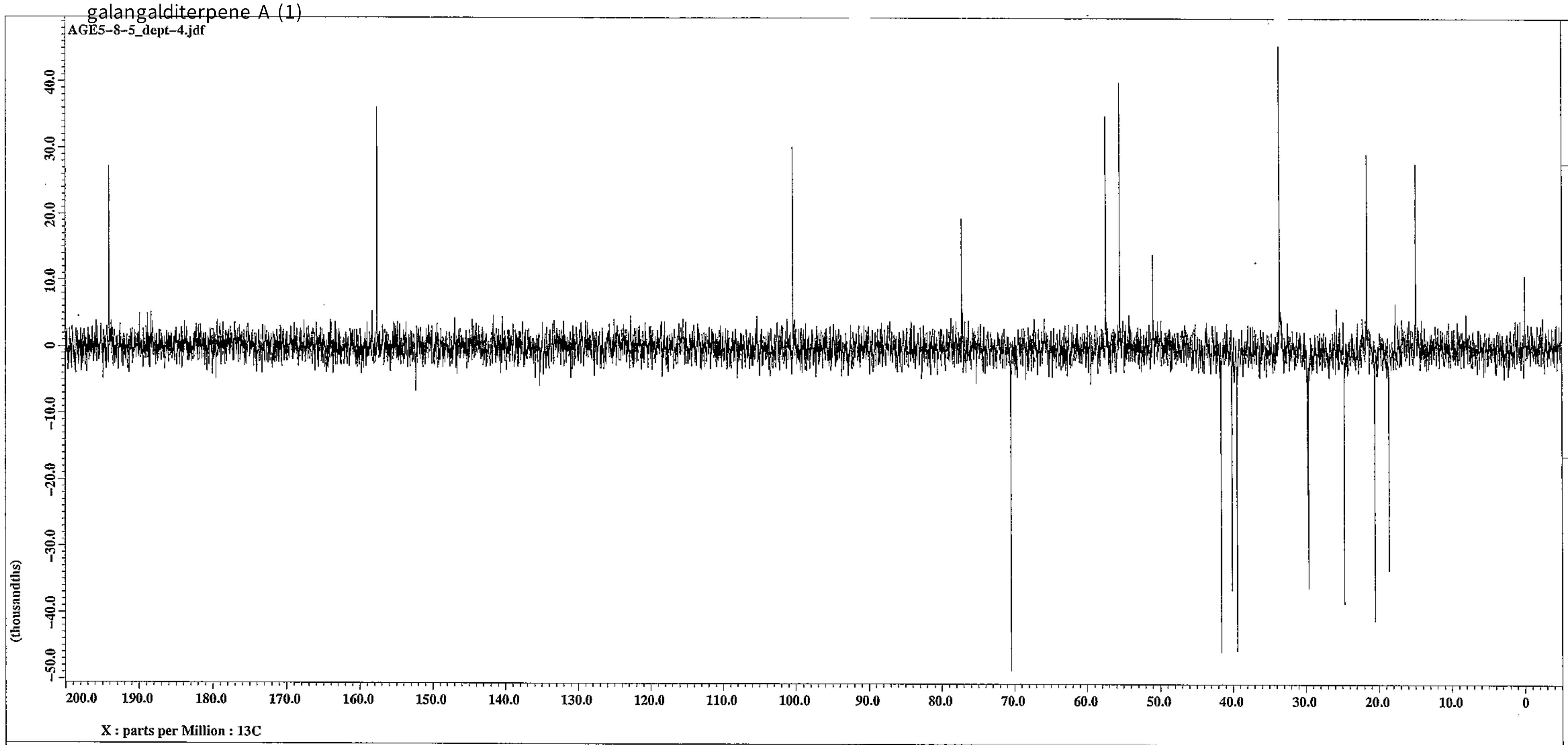

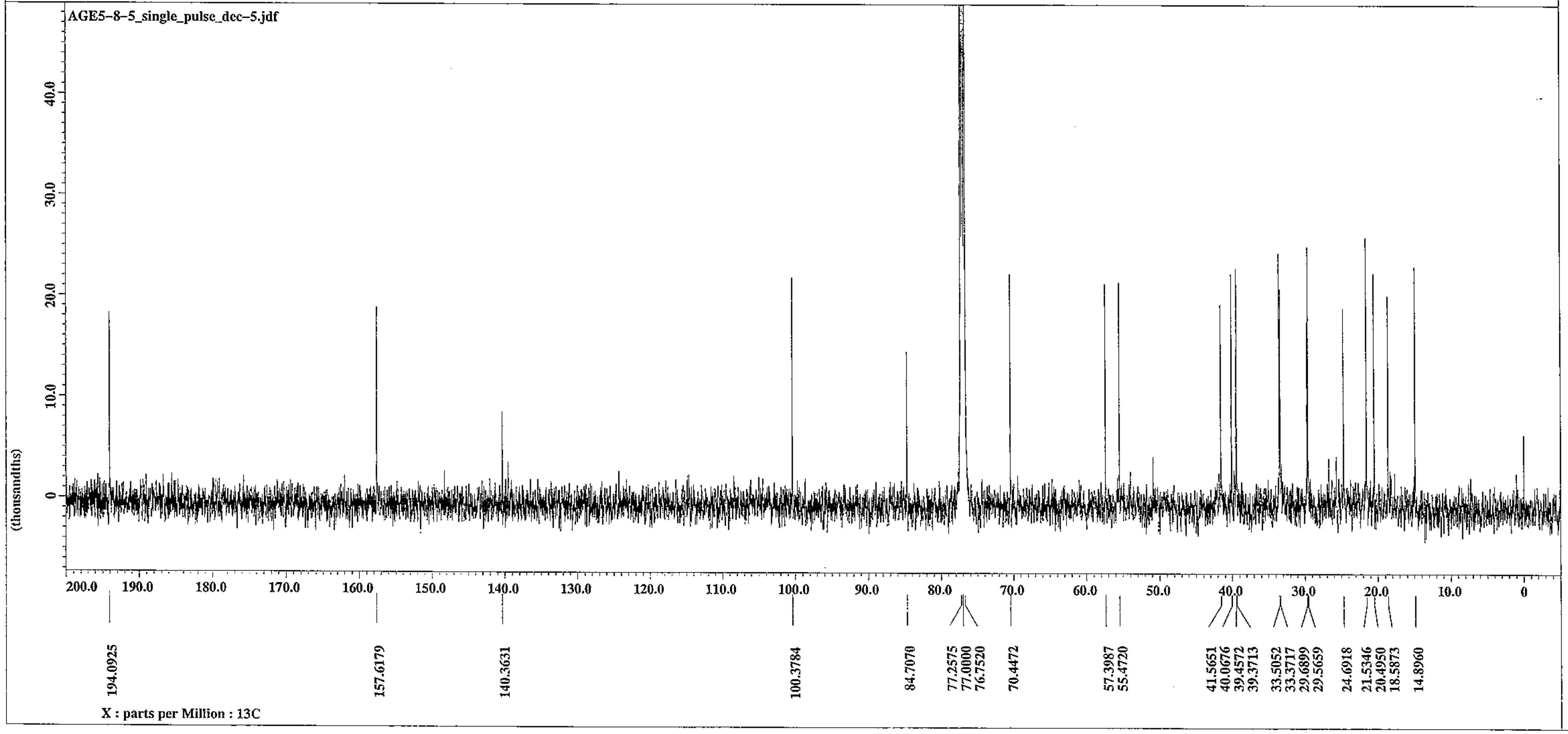

JEOL

---- PROCESSING PARAMETERS ----
dc_balance : 0 : FALSE
sexp : 2.0[Hz] : 0.0[s]
trapezoid3 : 0[%] : 80[%] : 100[%]
zerofill : 1
fft : 1 : TRUE : TRUE
machinephase
ppm

Filename = AGE5-8-5_single_pulse
Author = delta
Experiment = single_pulse_dec
Sample_id = 001
Solvent = CHLOROFORM-D
Creation_time = 14-FEB-2012 18:07:12
Revision_time = 15-FEB-2012 08:45:47
Current_time = 15-FEB-2012 08:47:01

Comment = single pulse decouple
Data_format = 1D COMPLEX
Dim_size = 26214
Dim_title = 13C
Dim_units = [ppm]
Dimensions = X
Site = ECA500
Spectrometer = JNM-ECA500

Field_strength = 11.7473579[T] (500[MH
X_acq_duration = 0.83361792[s]
X_domain = 13C
X_freq = 125.76529768[MHz]
X_offset = 100[ppm]
X_points = 32768
X_prescans = 4
X_resolution = 1.19959034[Hz]
X_sweep = 39.3081761[kHz]
Irr_domain = 1H
Irr_freq = 500.15991521[MHz]
Irr_offset = 5.0[ppm]
Clipped = FALSE
Mod_return = 1
Scans = 5000
Total_scans = 5000

X_90_width = 12[us]
X_acq_time = 0.83361792[s]
X_angle = 30[deg]
X_atn = 8.4[dB]
X_pulse = 4[us]
Irr_atn_dec = 20.84[dB]
Irr_atn_noe = 20.84[dB]
Irr_noise = WALTZ
Decoupling = TRUE
Initial_wait = 1[s]
Noe = TRUE
Noe_time = 2[s]
Recvr_gain = 58
Relaxation_delay = 2[s]
Repetition_time = 2.83361792[s]
Temp_get = 23.3[dC]

galangalditerpene A (1)

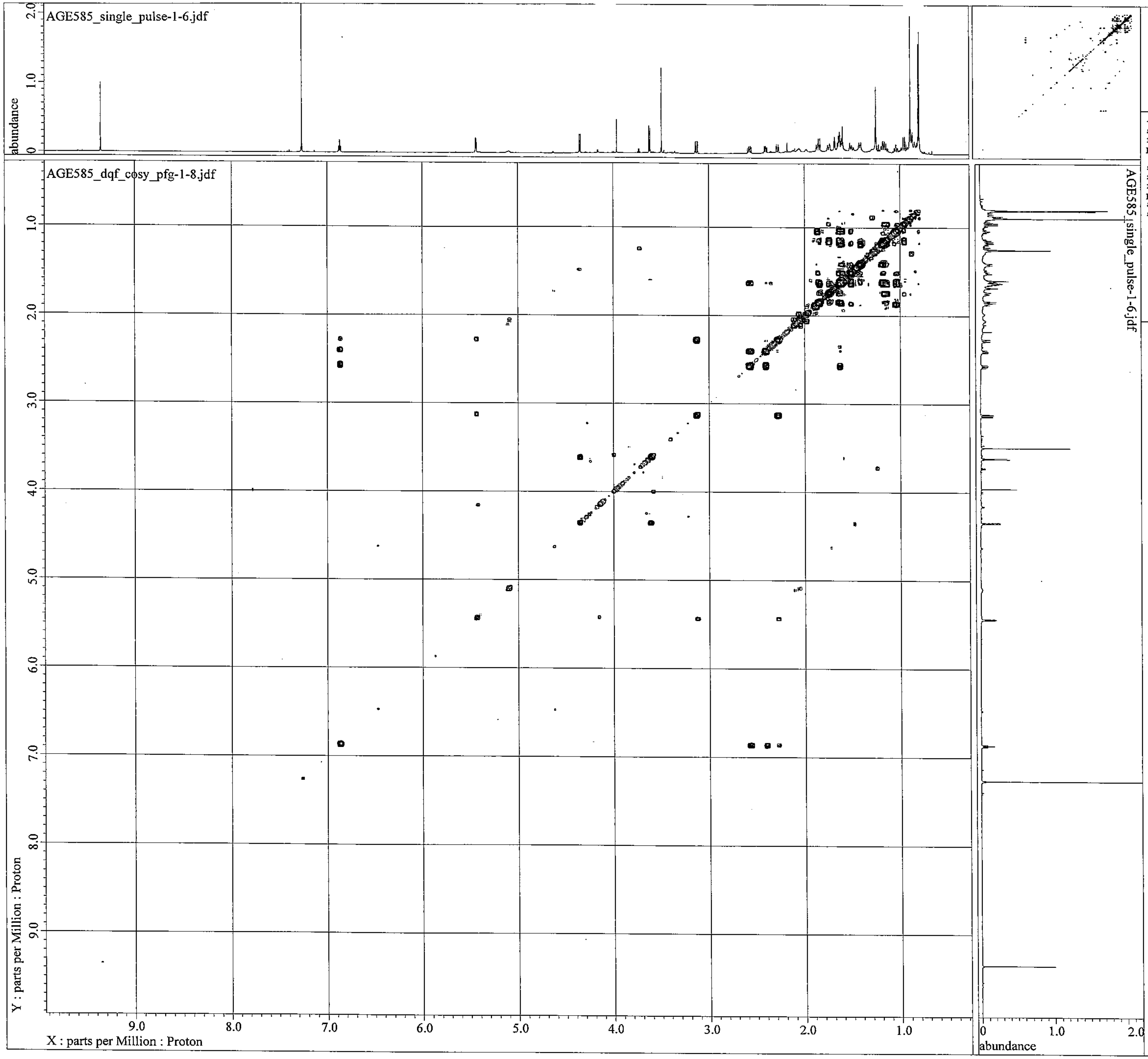


```
---- PROCESSING PARAMETERS ----
sinbell_auto
fft( 1, TRUE, TRUE )
ppm
[transpose]
sinbell_auto
zerofill( 4 )
fft( 1, TRUE, TRUE )
ppm
abs
symmetrize( Cosy, 24 )
[transpose]
```

```
Filename            = C:\Users\datum\Documents\J
Author              = delta
Experiment          = dqf_cosy_pfg.jxp
Sample_Id           = AGE585
Solvent             = CHLOROFORM-D
Creation_Time       = 15-FEB-2012 12:50:32
Revision_Time       = 15-FEB-2012 14:02:40
Current_Time        = 15-FEB-2012 14:04:24

Comment             = gradient absolute value DQ
Data_Format         = 2D REAL REAL
Dim_Size            = 1024, 1024
Dim_Title           = Proton Proton
Dim_Units           = [ppm] [ppm]
Dimensions          = X Y
Site                = JNM-ECA800
Spectrometer        = DELTA2_NMR

Field_Strength      = 18.79305132[T] (800[MHz])
X_Acq_Duration      = 0.1324032[s]
X_Domain            = 1H
X_Freq              = 800.14000039[MHz]
X_Offset            = 5.08312[ppm]
X_Points            = 1280
X_Prescans          = 4
X_Resolution        = 7.55268755[Hz]
X_Sweep             = 9.66744006[kHz]
X_Sweep_Clipped     = 7.73395205[kHz]
Y_Domain            = 1H
Y_Freq              = 800.14000039[MHz]
Y_Offset            = 5.08312[ppm]
Y_Points            = 256
Y_Prescans          = 0
Y_Resolution        = 30.21075019[Hz]
Y_Sweep             = 7.73395205[kHz]
Irr_Domain          = Proton
Irr_Freq            = 800.14000039[MHz]
Irr_Offset          = 5[ppm]
Tri_Domain          = Proton
Tri_Freq            = 800.14000039[MHz]
Tri_Offset          = 5[ppm]
Clipped             = FALSE
Scans               = 8
Total_Scans         = 2048

Relaxation_Delay    = 1.5[s]
Recvr_Gain          = 46
Temp_Get            = 19.8[dC]
X_Acq_Time          = 0.1324032[s]
X_Atn               = 1.3[dB]
X_Pulse             = 14[us]
Y_Acq_Time          = 33.1008[ms]
Irr_Mode            = Off
Tri_Mode            = Off
Dante_Presat        = FALSE
Grad_1              = 1[ms]
Grad_1_Amp          = 60[mT/m]
Grad_2              = 1[ms]
Grad_2_Amp          = 45[mT/m]
Grad_3              = 1[ms]
Grad_3_Amp          = 0.15[T/m]
Grad_Recover        = 0.1[ms]
Grad_Selection      = 4:3:10
Grad_Shape          = SINE
Initial_Wait        = 1[s]
Repetition_Time     = 1.6324032[s]
Scramble            = 1[ms]
T1                  = 1[us]
Zf_Factor           = 4
```

galangalditerpene A (1)

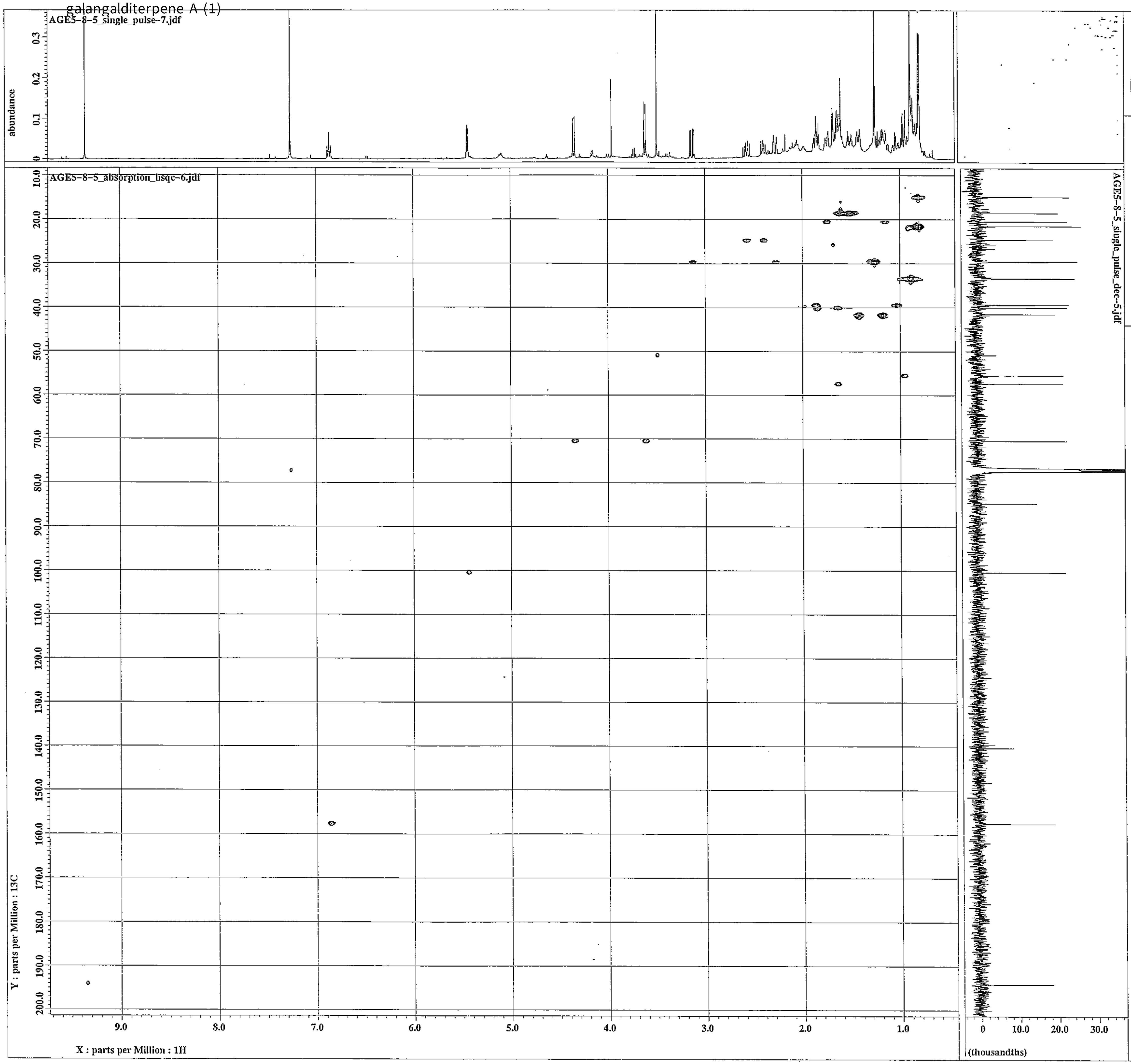


```
---- PROCESSING PARAMETERS ----
sexp : 10.0[Hz] : 0.0[s]
trapezoid3 : 0[%] : 80[%] : 100[%]
zerofill : 1
fft : 1 : TRUE : TRUE
ppm
machinephase
phase : -50 : 0 : 95.93023[%]
phase : 80 : -10 : 5.07525[ppm]
[transpose]
sexp : 50.0[Hz] : 0.0[s]
trapezoid3 : 0[%] : 80[%] : 100[%]
zerofill : 2
fft : 1 : TRUE : TRUE
ppm
machinephase
phase : 0 : 0 : 104.96322[ppm]
[transpose]
```

```
Filename            = AGE5-8-5_absorption_h
Author              = delta
Experiment          = hsqc_dec_phase_p
Sample_id           = 001
Solvent             = CHLOROFORM-D
Creation_time       = 14-FEB-2012 22:38:10
Revision_time       = 15-FEB-2012 08:53:01
Current_time        = 15-FEB-2012 08:53:49

Comment             = absorption_hsqc
Data_format         = 2D COMPLEX COMPLEX
Dim_size            = 819, 512
Dim_title           = 1H 13C
Dim_units           = [ppm] [ppm]
Dimensions          = X Y
Site                = ECA500
Spectrometer        = JNM-ECA500

Field_strength      = 11.7473579[T] (500[MH
X_acq_duration      = 0.176128[s]
X_domain            = 1H
X_freq              = 500.15991521[MHz]
X_offset            = 5.0639[ppm]
X_points            = 1024
X_prescans          = 4
X_resolution        = 5.67768895[Hz]
X_sweep             = 5.81395349[kHz]
Y_domain            = 13C
Y_freq              = 125.76529768[MHz]
Y_offset            = 104.77438[ppm]
Y_points            = 256
Y_prescans          = 0
Y_resolution        = 94.99635214[Hz]
Y_sweep             = 24.31906615[kHz]
Tri_domain          = 1H
Tri_freq            = 500.15991521[MHz]
Tri_offset          = 5.0[ppm]
Clipped             = FALSE
Mod_return          = 1
Scans               = 8
Total_scans         = 2048

X_acq_time          = 0.176128[s]
X_atn               = 2.4[dB]
X_pulse             = 12[us]
Y_acq_time          = 10.52672[ms]
Y_atn               = 8.4[dB]
Y_pl_correction     = 360
Y_pulse             = 12[us]
Irr_atn_dec         = 28[dB]
Irr_noise           = MPF10
Tri_mode            = Off
Composite_pulse     = FALSE
Dante_presat        = FALSE
Decoupling          = TRUE
Grad_1_amp          = 0.105[T/m]
Grad_2_amp          = 30[mT/m]
Initial_wait        = 1[s]
J_constant          = 140[Hz]
Purge               = 0[ms]
Recvr_gain          = 42
Relaxation_delay    = 1.5[s]
Repetition_time     = 1.676128[s]
T1                  = 0.88[us]
T1_cal1             = -9.4[us]
T1_cal2             = 0.88[us]
T1_cal3             = 11.16[us]
T1_cal4             = 21.44[us]
T1_cal5             = 31.72[us]
T1_cal6             = 42.0[us]
Temp_get            = 22.4[dC]
```

galangalditerpene A (1)

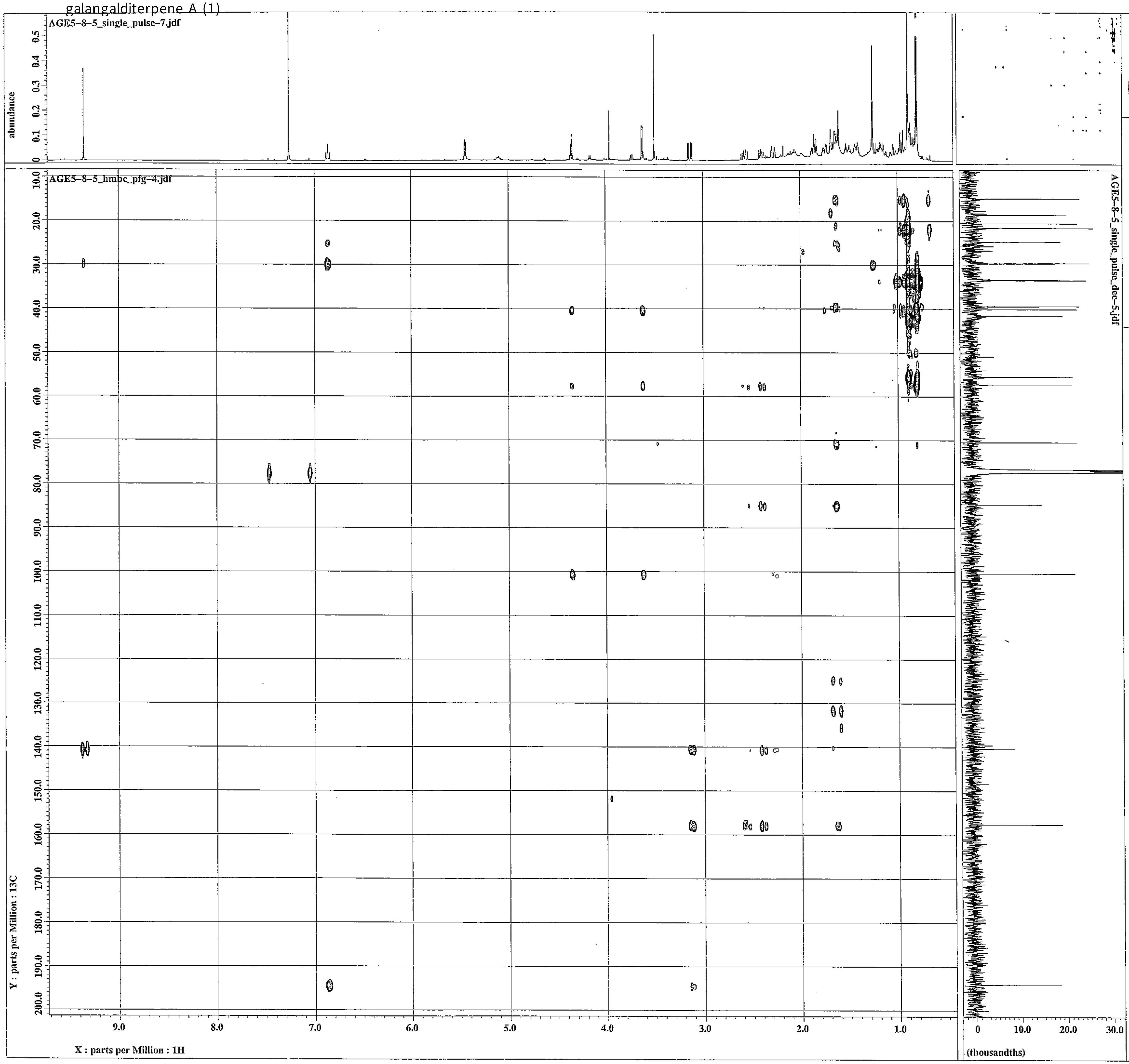


```
---- PROCESSING PARAMETERS ----
gauss : 5.0[Hz] : 0.0[s]
sinbell_auto
zerofill : 1
fft : 1 : TRUE : TRUE
ppm
[transpose]
sinbell4 : -60 : 160
trapezoid3 : 5[%] : 80[%] : 100[%]
zerofill : 2
fft : 1 : TRUE : TRUE
ppm
abs
[transpose]
```

```
Filename           = AGE5-8-5_hmbc_pfg-4.j
Author             = delta
Experiment         = hmbc_pfg.ex2
Sample_id          = 001
Solvent            = CHLOROFORM-D
Creation_time      = 15-FEB-2012 03:00:58
Revision_time      = 15-FEB-2012 08:59:18
Current_time       = 15-FEB-2012 09:00:02

Comment            = gradient enhanced HMB
Data_format        = 2D REAL REAL
Dim_size           = 1638, 512
Dim_title          = 1H 13C
Dim_units          = [ppm] [ppm]
Dimensions         = X Y
Site               = ECA500
Spectrometer       = JNM-ECA500

Field_strength     = 11.7473579[T] (500[MH
X_acq_duration     = 0.352256[s]
X_domain           = 1H
X_freq             = 500.15991521[MHz]
X_offset           = 5.0639[ppm]
X_points           = 2048
X_prescans         = 4
X_resolution       = 2.83884448[Hz]
X_sweep            = 5.81395349[kHz]
Y_domain           = 13C
Y_freq             = 125.76529768[MHz]
Y_offset           = 104.77438[ppm]
Y_points           = 256
Y_prescans         = 0
Y_resolution       = 94.99635214[Hz]
Y_sweep            = 24.31906615[kHz]
Tri_domain         = 1H
Tri_freq           = 500.15991521[MHz]
Tri_offset         = 5.0[ppm]
Clipped            = FALSE
Mod_return         = 1
Scans              = 32
Total_scans        = 8192

X_acq_time         = 0.352256[s]
X_atn              = 2.4[dB]
X_pulse            = 12[us]
Y_acq_time         = 10.52672[ms]
Y_atn              = 8.4[dB]
Y_pulse            = 12[us]
Tri_mode           = Off
Dante_presat       = FALSE
Delta              = 62.5[ms]
Grad_1             = 1[ms]
Grad_1_amp         = 0.18[T/m]
Grad_2             = 1[ms]
Grad_2_amp         = 0.18[T/m]
Grad_3             = 1[ms]
Grad_3_amp         = 90.54325956[mT/m]
Grad_recover       = 0.1[ms]
Grad_selection     = 13C = 1.988:1.98
Grad_shape         = SINE
Initial_wait       = 1[s]
J_constant         = 140[Hz]
Long_range_j       = 8[Hz]
Recvr_gain         = 42
Refocus_comp       = 2.57142857[ms]
Relaxation_delay   = 1.5[s]
Repetition_time    = 1.852256[s]
T1                 = 1[us]
Temp1              = 1.988
Temp_get           = 22[dC]
```

galangalditerpene A (1)

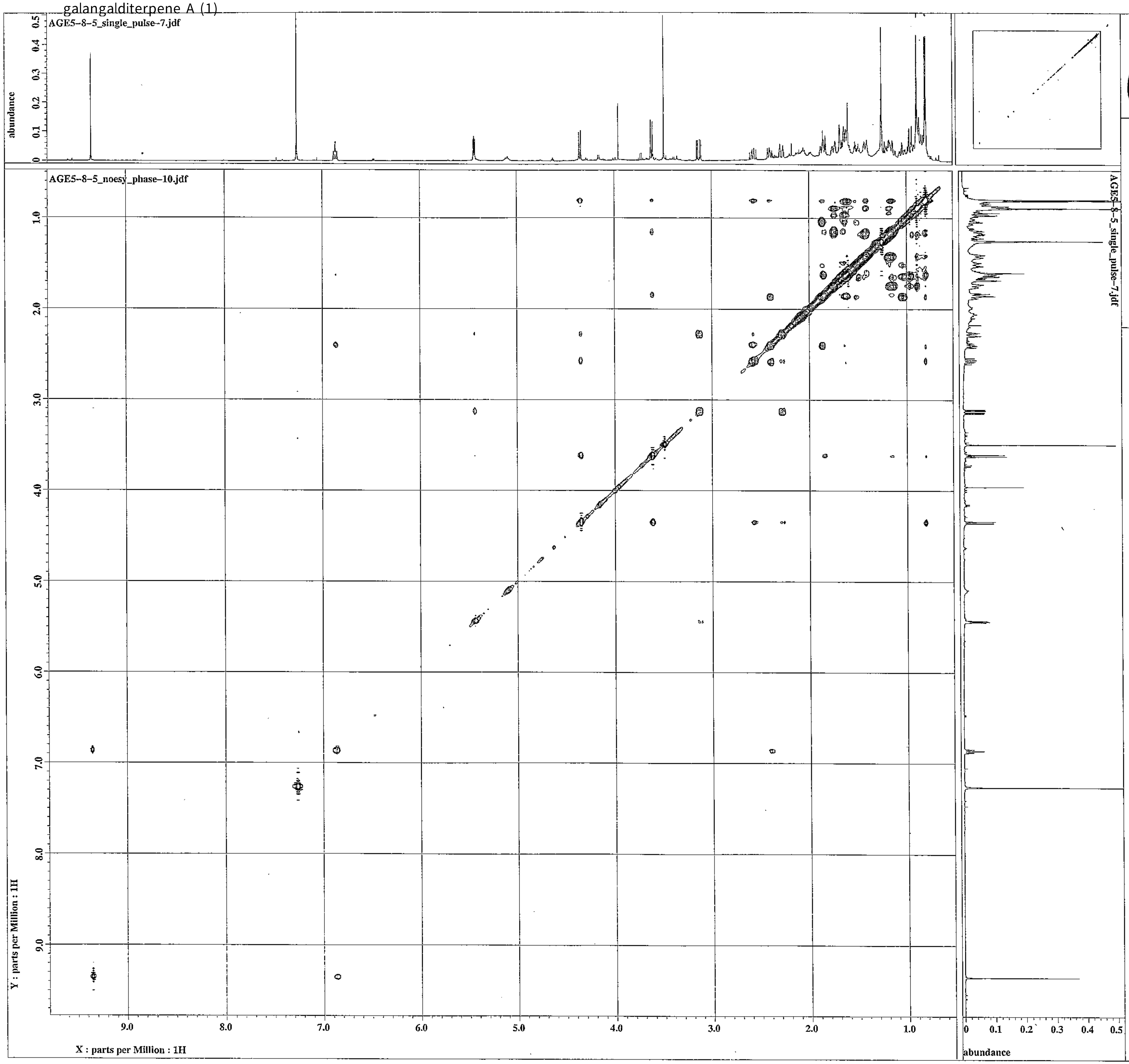


```
---- PROCESSING PARAMETERS ----
sexp : 5.0[Hz] : 0.0[s]
trapezoid3 : 0[%] : 80[%] : 100[%]
zerofill : 1
fft : 1 : TRUE : TRUE
ppm
machinephase
phase : -1.25 : 0 : 85.78384[%]
phase : 1 : 0 : 5.07856[ppm]
[transpose]
sexp : 5.0[Hz] : 0.0[s]
trapezoid3 : 0[%] : 80[%] : 100[%]
zerofill : 2
fft : 1 : TRUE : TRUE
ppm
machinephase
phase : 0 : 0 : 5.07562[ppm]
[transpose]
```

```
Filename            = AGE5-8-5_noesy_phase-
Author              = delta
Experiment          = noesy_phase.ex2
Sample_id           = 001
Solvent             = CHLOROFORM-D
Creation_time       = 15-FEB-2012 09:32:00
Revision_time       = 15-FEB-2012 10:01:35
Current_time        = 15-FEB-2012 10:03:11

Comment             = phase sensitive noesy
Data_format         = 2D COMPLEX COMPLEX
Dim_size            = 819, 512
Dim_title           = 1H 1H
Dim_units           = [ppm] [ppm]
Dimensions          = X Y
Site                = ECA500
Spectrometer        = JNM-ECA500

Field_strength      = 11.7473579[T] (500[MH
X_acq_duration      = 0.1363968[s]
X_domain            = 1H
X_freq              = 500.15991521[MHz]
X_offset            = 5.0639[ppm]
X_points            = 1024
X_prescans          = 4
X_resolution        = 7.3315503[Hz]
X_sweep             = 7.50750751[kHz]
Y_domain            = 1H
Y_freq              = 500.15991521[MHz]
Y_offset            = 5.0639[ppm]
Y_points            = 256
Y_prescans          = 0
Y_resolution        = 23.44687875[Hz]
Y_sweep             = 6.00240096[kHz]
Irr_domain          = 1H
Irr_freq            = 500.15991521[MHz]
Irr_offset          = 5.0[ppm]
Tri_domain          = 1H
Tri_freq            = 500.15991521[MHz]
Tri_offset          = 5.0[ppm]
Clipped             = FALSE
Mod_return          = 1
Scans               = 16
Total_scans         = 4096

X_acq_time          = 0.1363968[s]
X_atn               = 2.4[dB]
X_pulse             = 12[us]
Y_acq_time          = 42.6496[ms]
Y_p0_correction     = 0
Y_p1_correction     = 180
Irr_mode            = Off
Tri_mode            = Off
Dante_presat        = FALSE
Initial_wait        = 1[s]
Mix_time            = 0.7[s]
Recvr_gain          = 42
Relaxation_delay    = 2[s]
Repetition_time     = 2.1363968[s]
Scramble            = 1[ms]
T1                  = 67.94[us]
T1_cal1             = 67.94[us]
T1_cal2             = 0.15124[ms]
T1_cal3             = 0.23454[ms]
T1_cal4             = 0.31784[ms]
T1_cal5             = 0.40114[ms]
T1_cal6             = 0.48444[ms]
Temp_get            = 22[dC]
```

galangalditerpene B (2)

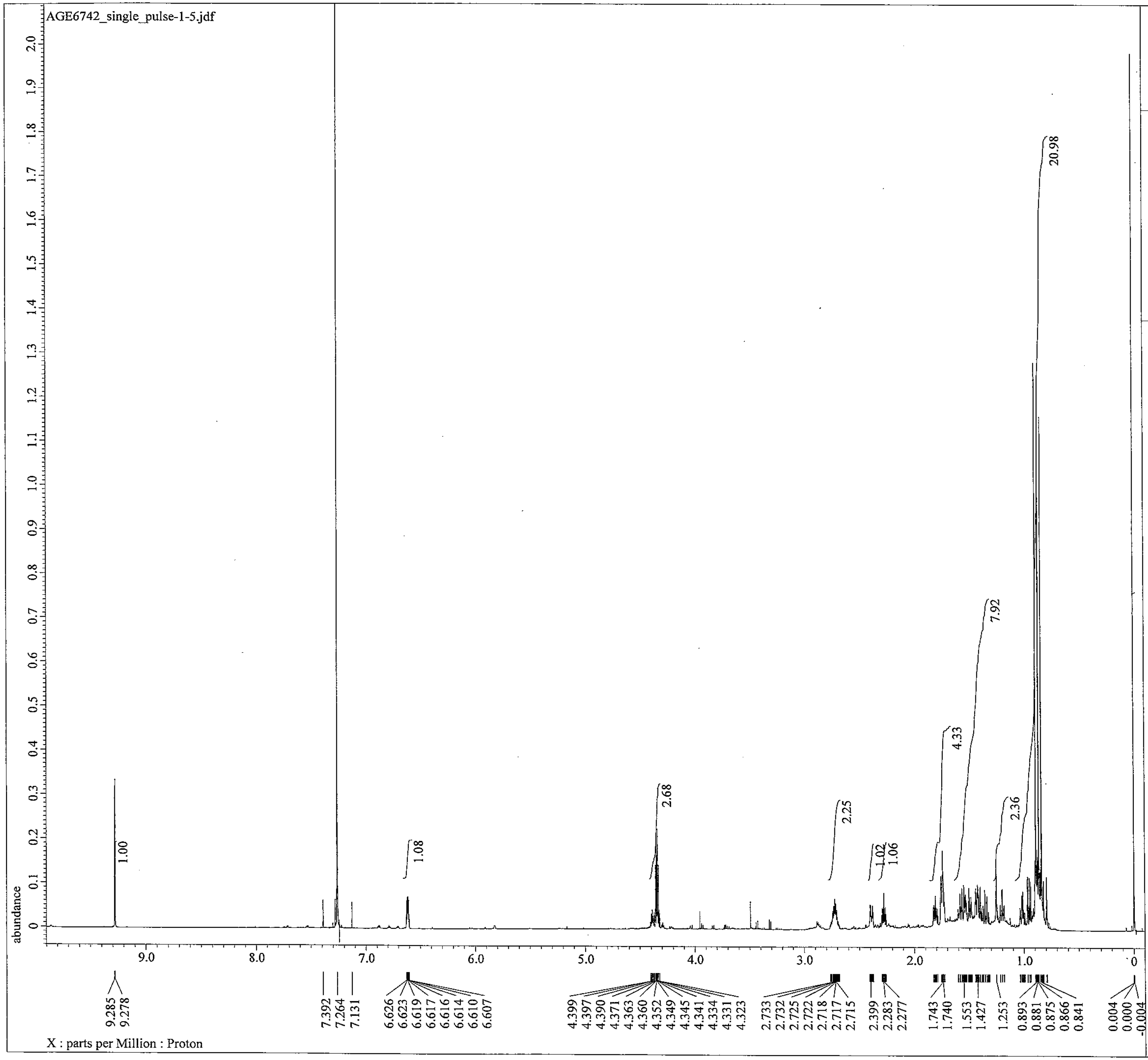


```
---- PROCESSING PARAMETERS ----
dc_balance( 0, FALSE )
sexp( 0.2[Hz], 0.0[s] )
trapezoid( 0[%], 0[%], 80[%], 100[%] )
zerofill( 1 )
fft( 1, TRUE, TRUE )
machinephase
ppm
phase( -4, 1, 35.02327[%] )

以下に由来:: AGE6742_single_pulse-1-1.jdf
```

```
Filename           = C:\Users\delta\Documents\J
Author             = delta
Experiment         = single_pulse.jxp
Sample_Id          = AGE6742
Solvent            = CHLOROFORM-D
Creation_Time      = 20-SEP-2013 14:22:42
Revision_Time      = 20-SEP-2013 15:46:00
Current_Time       = 20-SEP-2013 15:46:11

Comment            = single_pulse
Data_Format        = 1D COMPLEX
Dim_Size           = 52429
Dim_Title          = Proton
Dim_Units          = [ppm]
Dimensions         = X
Site               = JNM-ECA800
Spectrometer       = DELTA2_NMR

Field_Strength     = 18.79305132[T] (800[MHz])
X_Acq_Duration     = 4.36207616[s]
X_Domain           = 1H
X_Freq             = 800.14000039[MHz]
X_Offset           = 5[ppm]
X_Points           = 65536
X_Prescans         = 1
X_Resolution       = 0.22924863[Hz]
X_Sweep            = 15.02403846[kHz]
X_Sweep_Clipped    = 12.01923077[kHz]
Irr_Domain         = Proton
Irr_Freq           = 800.14000039[MHz]
Irr_Offset         = 5[ppm]
Tri_Domain         = Proton
Tri_Freq           = 800.14000039[MHz]
Tri_Offset         = 5[ppm]
Clipped            = FALSE
Scans              = 16
Total_Scans        = 16

Relaxation_Delay   = 5[s]
Recvr_Gain         = 46
Temp_Get           = 22[dC]
X_90_Width         = 15.5[us]
X_Acq_Time         = 4.36207616[s]
X_Angle            = 45[deg]
X_Atn              = 3.2[dB]
X_Pulse            = 7.75[us]
Irr_Mode           = Off
Tri_Mode           = Off
Dante_Presat       = FALSE
Initial_Wait       = 1[s]
Repetition_Time    = 9.36207616[s]
```

galangalditerpene B (2)

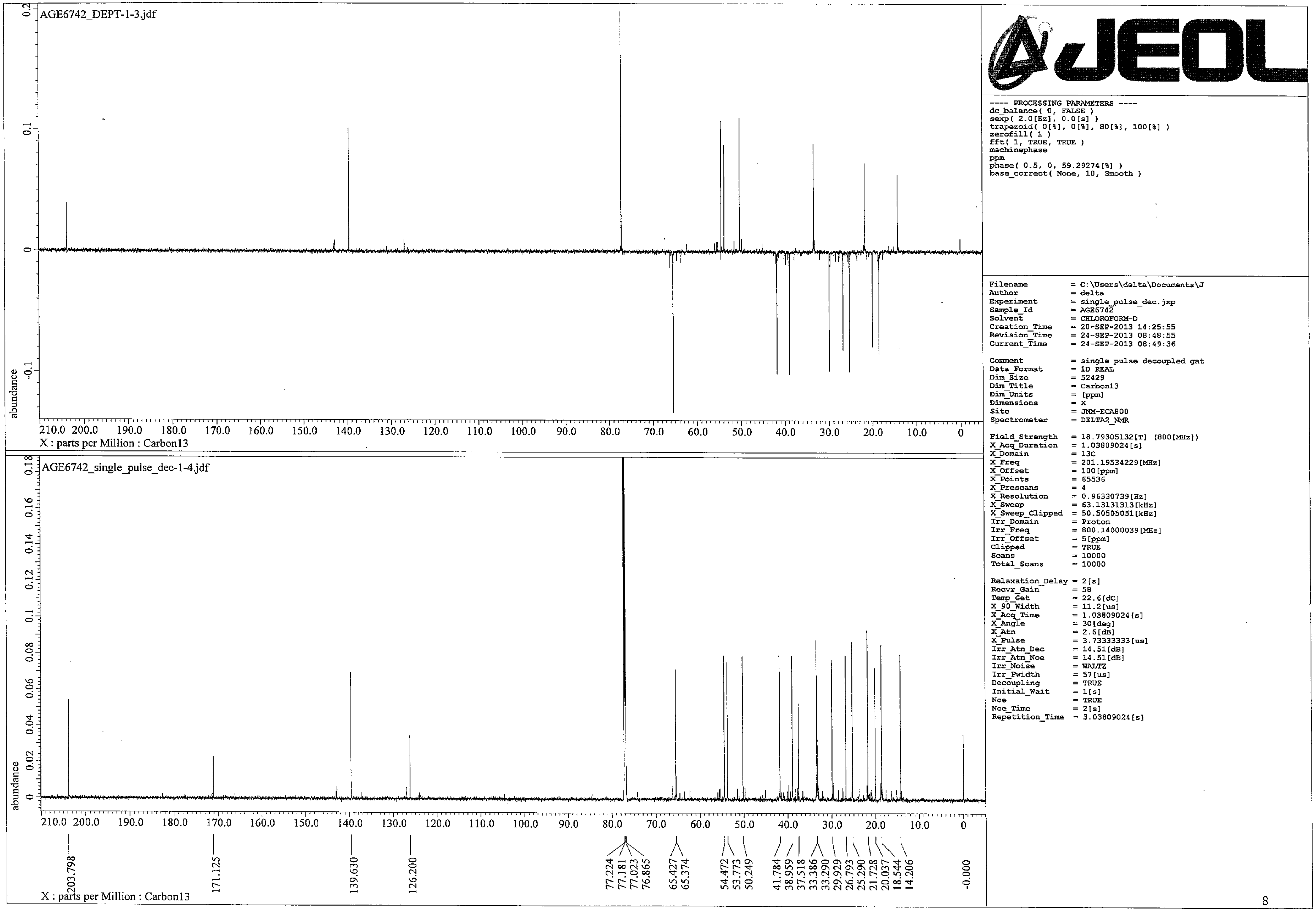

galangalditerpene B (2)

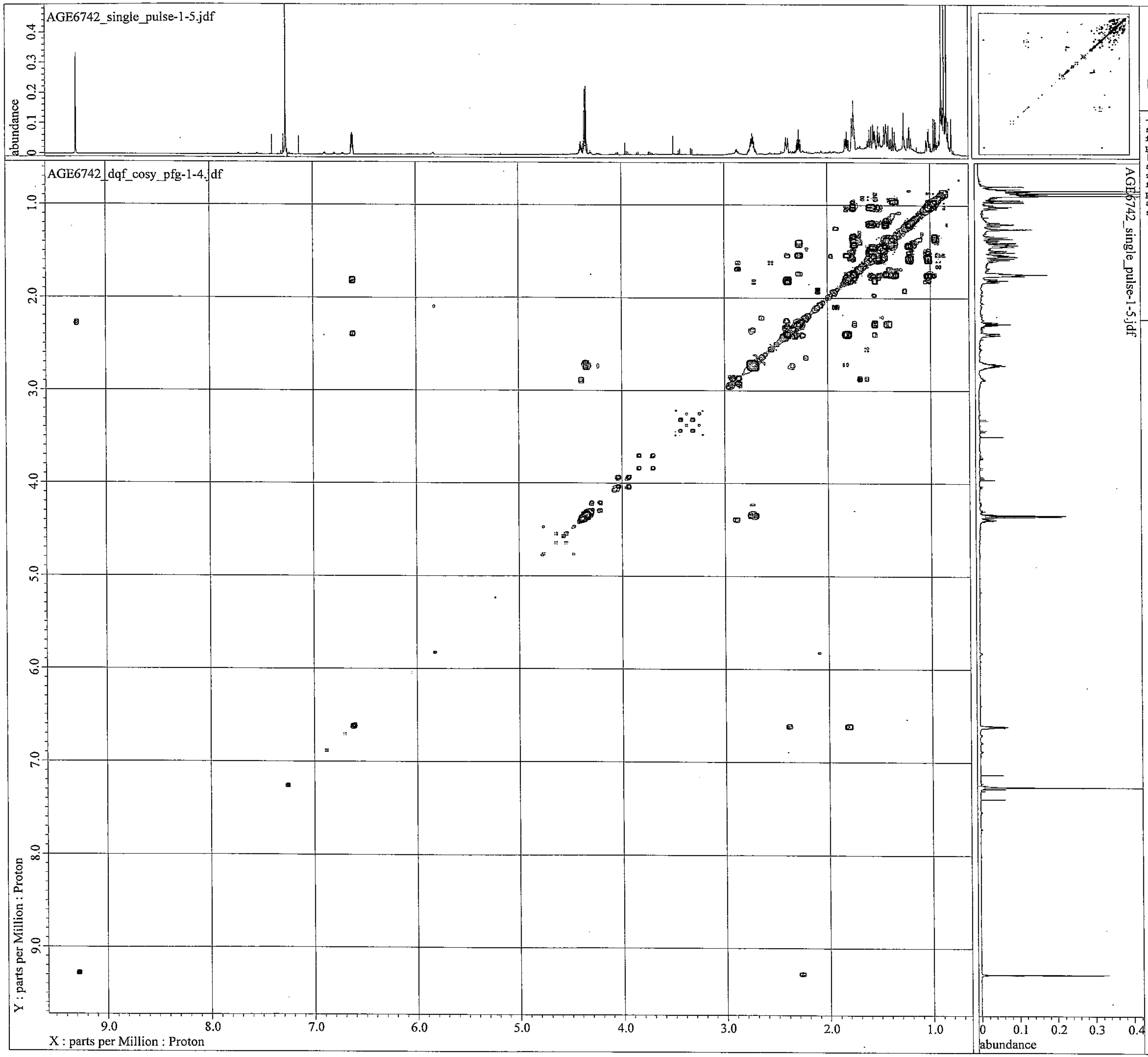


```
---- PROCESSING PARAMETERS ----
sinbell_auto
fft( 1, TRUE, TRUE )
ppm
[transpose]
sinbell_auto
zerofill( 4 )
fft( 1, TRUE, TRUE )
ppm
abs
symmetrize( Cosy, 24 )
[transpose]
```

```
Filename            = C:\Users\delta\Documents\J
Author              = delta
Experiment          = dqf_cosy_pfg.jxp
Sample_Id           = AGE6742
Solvent             = CHLOROFORM-D
Creation_Time       = 21-SEP-2013 01:55:58
Revision_Time       = 24-SEP-2013 08:51:30
Current_Time        = 24-SEP-2013 08:52:23

Comment             = gradient absolute value DQ
Data_Format         = 2D REAL REAL
Dim_Size            = 1024, 1024
Dim_Title           = Proton Proton
Dim_Units           = [ppm] [ppm]
Dimensions          = X Y
Site                = JNM-ECA800
Spectrometer        = DELTA2_NMR

Field_Strength      = 18.79305132[T] (800[MHz])
X_Acq_Duration      = 0.1278976[s]
X_Domain            = 1H
X_Freq              = 800.14000039[MHz]
X_Offset            = 5[ppm]
X_Points            = 1280
X_Prescans          = 4
X_Resolution        = 7.818755[Hz]
X_Sweep             = 10.00800641[kHz]
X_Sweep_Clipped     = 8.00640512[kHz]
Y_Domain            = 1H
Y_Freq              = 800.14000039[MHz]
Y_Offset            = 5[ppm]
Y_Points            = 256
Y_Prescans          = 0
Y_Resolution        = 31.2600032[Hz]
Y_Sweep             = 8.00256082[kHz]
Irr_Domain          = Proton
Irr_Freq            = 800.14000039[MHz]
Irr_Offset          = 5[ppm]
Tri_Domain          = Proton
Tri_Freq            = 800.14000039[MHz]
Tri_Offset          = 5[ppm]
Clipped             = FALSE
Scans               = 8
Total_Scans         = 2048

Relaxation_Delay    = 1.5[s]
Recvr_Gain          = 44
Temp_Get            = 21.9[dC]
X_Acq_Time          = 0.1278976[s]
X_Atn               = 3.2[dB]
X_Pulse             = 15.5[us]
Y_Acq_Time          = 31.98976[ms]
Irr_Mode            = Off
Tri_Mode            = Off
Dante_Presat        = FALSE
Grad_1              = 1[ms]
Grad_1_Amp          = 60[mT/m]
Grad_2              = 1[ms]
Grad_2_Amp          = 45[mT/m]
Grad_3              = 1[ms]
Grad_3_Amp          = 0.15[T/m]
Grad_Recover        = 0.1[ms]
Grad_Selection      = 4:3:10
Grad_Shape          = SINE
Initial_Wait        = 1[s]
Repetition_Time     = 1.6278976[s]
Scramble            = 1[ms]
T1                  = 1[us]
Zf_Factor           = 4
```

galangalditerpene B (2)

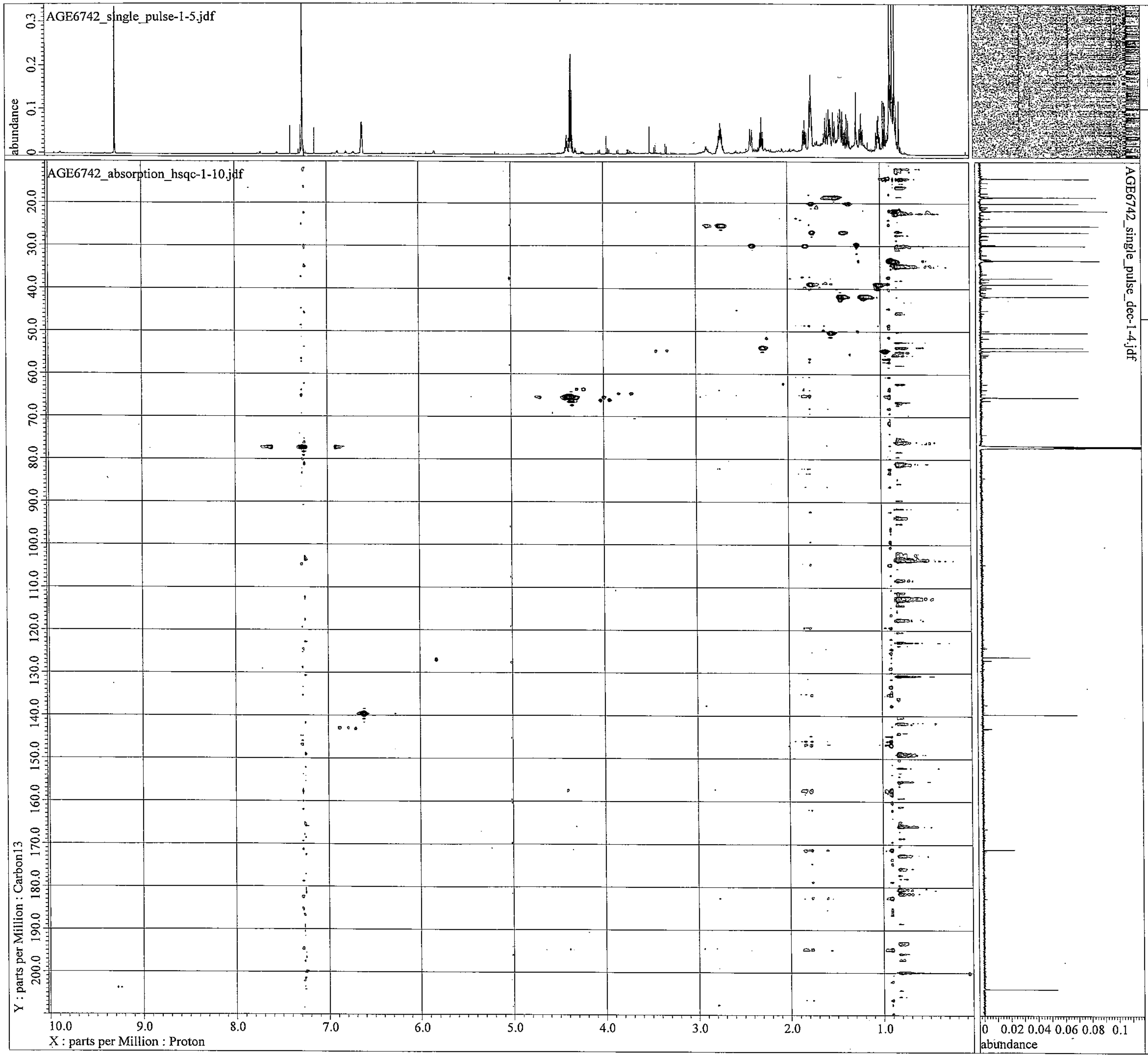


```
---- PROCESSING PARAMETERS ----
sexp( 10.0[Hz], 0.0[s] )
trapezoid( 0[%], 0[%], 80[%], 100[%] )
zerofill( 1 )
fft( 1, TRUE, TRUE )
ppm
machinephase
phase( -24.48151, 0, 92.05379[%] )
[transpose]
sexp( 50.0[Hz], 0.0[s] )
trapezoid( 0[%], 0[%], 80[%], 100[%] )
zerofill( 2 )
fft( 1, TRUE, TRUE )
ppm
machinephase
[transpose]
```

```
Filename            = C:\Users\delta\Documents\J
Author              = delta
Experiment          = hsqc_dec_phase_pfgzz.jxp
Sample_Id           = AGE6742
Solvent             = CHLOROFORM-D
Creation_Time       = 21-SEP-2013 02:52:54
Revision_Time       = 24-SEP-2013 08:56:55
Current_Time        = 24-SEP-2013 08:58:20

Comment             = absorption_hsqc
Data_Format         = 2D COMPLEX COMPLEX
Dim_Size            = 819, 512
Dim_Title           = Proton Carbon13
Dim_Units           = [ppm] [ppm]
Dimensions          = X Y
Site                = JNM-ECA800
Spectrometer        = DELTA2_NMR

Field_Strength      = 18.79305132[T] (800[MHz])
X_Acq_Duration      = 0.10231808[s]
X_Domain            = 1H
X_Freq              = 800.14000039[MHz]
X_Offset            = 5[ppm]
X_Points            = 1024
X_Prescans          = 4
X_Resolution        = 9.77344376[Hz]
X_Sweep             = 10.00800641[kHz]
X_Sweep_Clipped     = 8.00640512[kHz]
Y_Domain            = 13C
Y_Freq              = 201.19534229[MHz]
Y_Offset            = 110[ppm]
Y_Points            = 256
Y_Prescans          = 0
Y_Resolution        = 157.25644122[Hz]
Y_Sweep             = 40.25764895[kHz]
Tri_Domain          = Proton
Tri_Freq            = 800.14000039[MHz]
Tri_Offset          = 5[ppm]
Clipped             = FALSE
Scans               = 8
Total_Scans         = 2048

Relaxation_Delay    = 1.5[s]
Recvr_Gain          = 44
Temp_Get            = 21.9[dC]
X_Acq_Time          = 0.10231808[s]
X_Atn               = 3.2[dB]
X_Pulse             = 15.5[us]
Y_Acq_Time          = 6.35904[ms]
Y_Atn               = 2.6[dB]
Y_P1_Correction     = 720
Y_Pulse             = 11.2[us]
Irr_Atn_Dec         = 20.985[dB]
Irr_Noise           = MPF10
Irr_Pwidth          = 93[us]
Tri_Mode            = Off
Composite_Pulse     = FALSE
Dante_Presat        = FALSE
Decoupling          = TRUE
Grad_1_Amp          = 0.105[T/m]
Grad_2_Amp          = 30[mT/m]
Initial_Wait        = 1[s]
J_Constant          = 140[Hz]
Phase_Qd            = {{0, 180}, {90, 270}}
Purge               = 0[ms]
Repetition_Time     = 1.60231808[s]
T1                  = 2.172[us]
T1_Cal1             = -16.458[us]
T1_Cal2             = -10.248[us]
T1_Cal3             = -4.038[us]
T1_Cal4             = 2.172[us]
T1_Cal5             = 8.382[us]
```

galangalditerpene B (2)

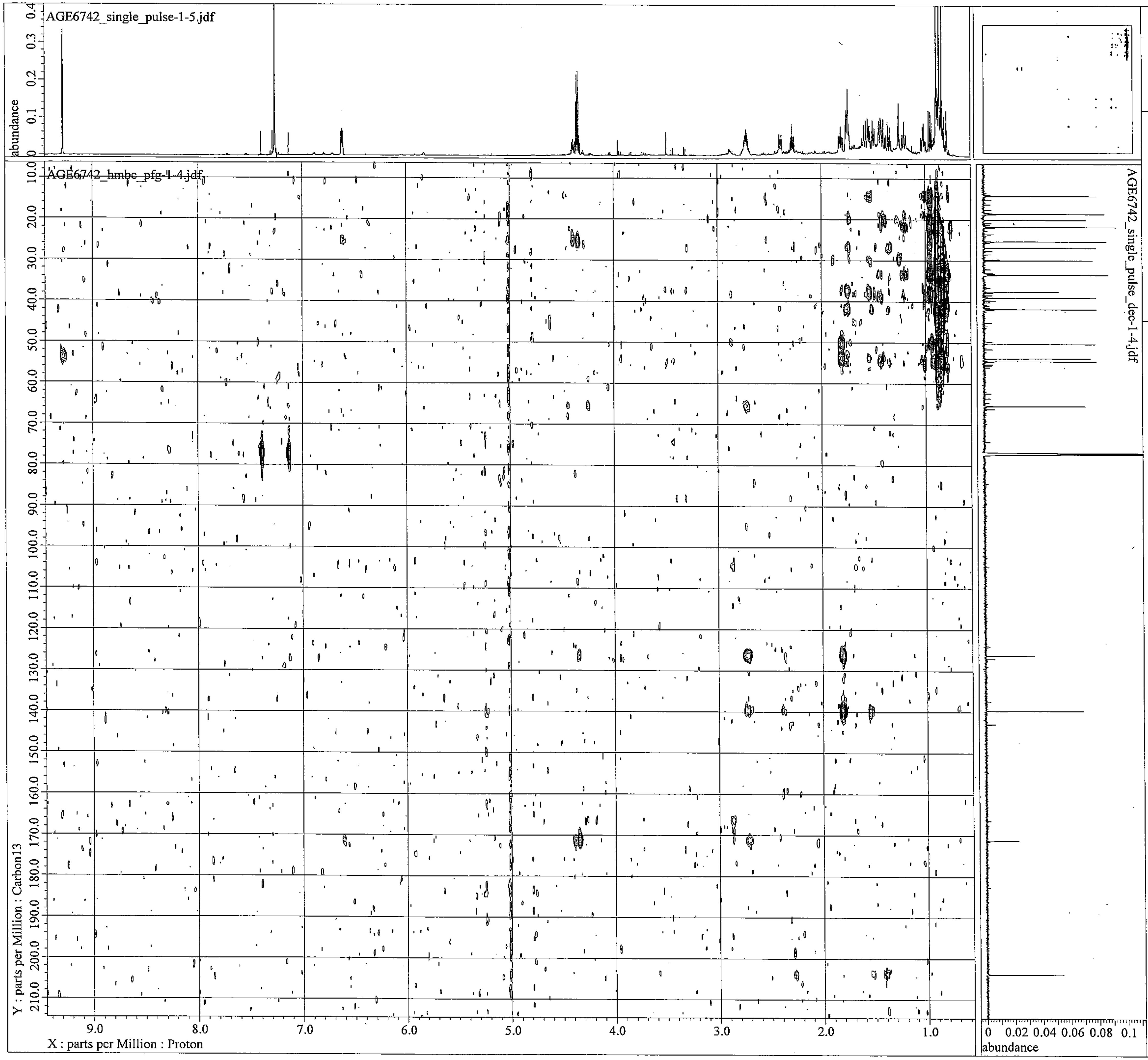

---- PROCESSING PARAMETERS ----
gauss(5.0[Hz], 0.0[s])
sinbell_auto
zerofill(1)
fft(1, TRUE, TRUE)
ppm
[transpose]
sinbell4(-60, 160)
trapezoid(0[%], 5[%], 80[%], 100[%])
zerofill(2)
fft(1, TRUE, TRUE)
ppm
abs
[transpose]

Filename = C:\Users\delta\Documents\J
Author = delta
Experiment = hmbc_pfg.jxp
Sample_Id = AGE6742
Solvent = CHLOROFORM-D
Creation_Time = 21-SEP-2013 04:43:50
Revision_Time = 24-SEP-2013 09:01:49
Current_Time = 24-SEP-2013 09:03:13

Comment = gradient enhanced HMBC
Data_Format = 2D REAL REAL
Dim_Size = 512, 1638
Dim_Title = Carbon13 Proton
Dim_Units = [ppm] [ppm]
Dimensions = X Y
Site = JNM-ECA800
Spectrometer = DELTA2_NMR

Field_Strength = 18.79305132[T] (800[MHz])
X_Acq_Duration = 0.20463616[s]
X_Domain = 1H
X_Freq = 800.14000039[MHz]
X_Offset = 5[ppm]
X_Points = 2048
X_Prescans = 4
X_Resolution = 4.88672188[Hz]
X_Sweep = 10.00800641[kHz]
X_Sweep_Clipped = 8.00640512[kHz]
Y_Domain = 13C
Y_Freq = 201.19534229[MHz]
Y_Offset = 100[ppm]
Y_Points = 256
Y_Prescans = 0
Y_Resolution = 196.49144869[Hz]
Y_Sweep = 50.30181087[kHz]
Tri_Domain = Proton
Tri_Freq = 800.14000039[MHz]
Tri_Offset = 5[ppm]
Clipped = FALSE
Scans = 32
Total_Scans = 8192

Relaxation_Delay = 1.5[s]
Recvr_Gain = 44
Temp_Get = 21.9[dC]
X_Acq_Time = 0.20463616[s]
X_Atn = 3.2[dB]
X_Pulse = 15.5[us]
Y_Acq_Time = 5.08928[ms]
Y_Atn = 2.6[dB]
Y_Pulse = 11.2[us]
Tri_Mode = Off
Dante_Presat = FALSE
Delta = 62.5[ms]
Delta_1 = 3.83288616[ms]
Delta_2 = 3.43642612[ms]
Delta_3 = 3.11429461[ms]
Grad_1 = 1[ms]
Grad_1_Amp = 0.18[T/m]
Grad_2 = 1[ms]
Grad_2_Amp = 0.18[T/m]
Grad_3 = 1[ms]
Grad_3_Amp = 90.54325956[mT/m]
Grad_Lpf = 1[ms]
Grad_Lpf1_Amp = 45[mT/m]
Grad_Lpf2_Amp = -25.71428571[mT/m]
Grad_Lpf3_Amp = -12.85714286[mT/m]
Grad_Lpf4_Amp = -6.42857143[mT/m]
Grad_Recover = 0.1[ms]
Grad_Selection = 13C = 1.988:1.988:1
Grad_Shape = SINE
Initial_Wait = 1[s]

galangalditerpene B (2)

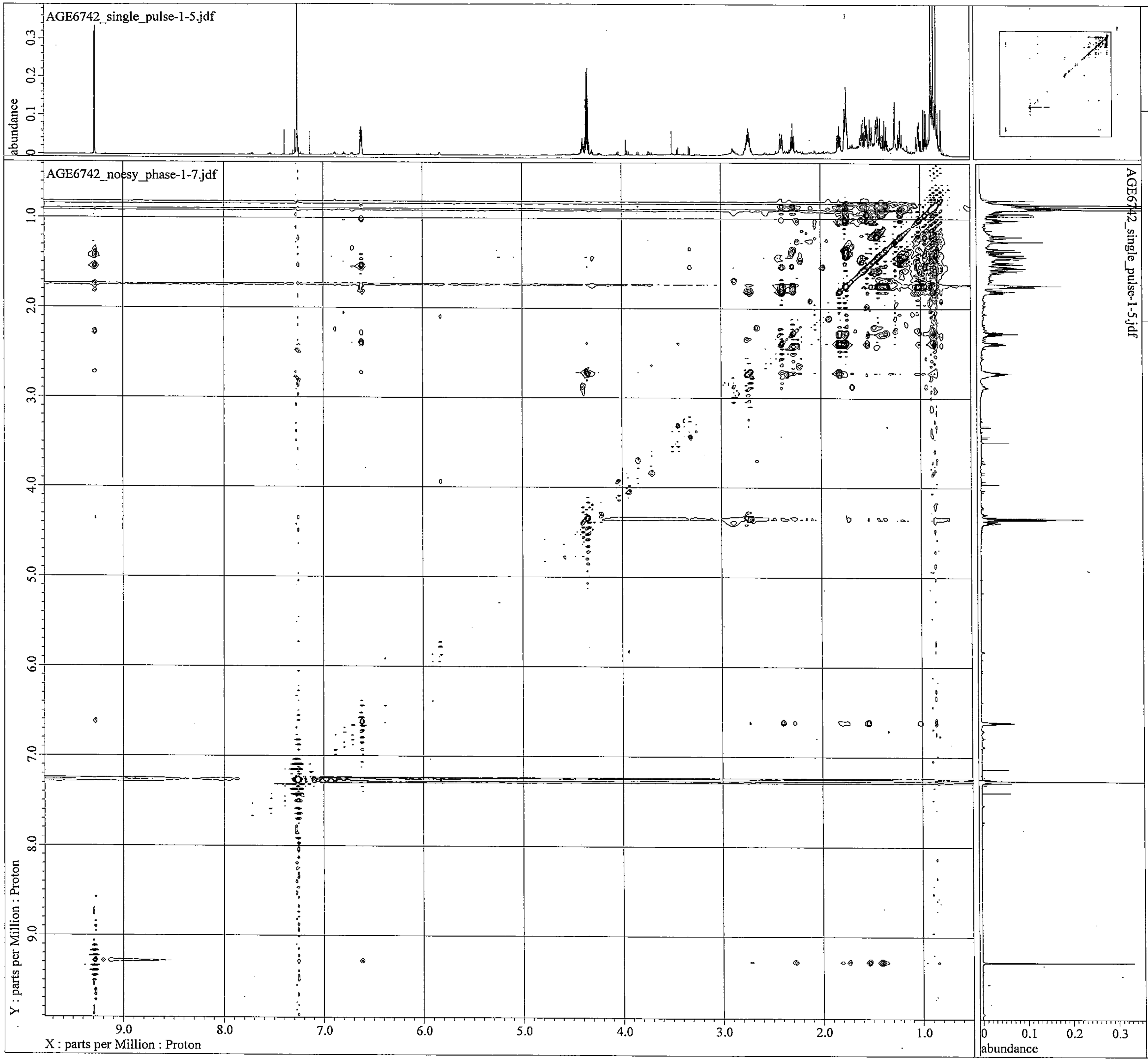


```
---- PROCESSING PARAMETERS ----
sexp( 5.0[Hz], 0.0[s] )
trapezoid( 0[%], 0[%], 80[%], 100[%] )
zerofill( 1 )
fft( 1, TRUE, TRUE )
ppm
machinephase
phase( 9.78358, -0.89816, 34.10758[%] )
[transpose]
sexp( 5.0[Hz], 0.0[s] )
trapezoid( 0[%], 0[%], 80[%], 100[%] )
zerofill( 2 )
fft( 1, TRUE, TRUE )
ppm
machinephase
[transpose]
```

```
Filename           = C:\Users\delta\Documents\J
Author             = delta
Experiment         = noesy_phase.jxp
Sample_Id          = AGE6742
Solvent            = CHLOROFORM-D
Creation_Time      = 21-SEP-2013 08:46:30
Revision_Time      = 24-SEP-2013 09:14:03
Current_Time       = 24-SEP-2013 09:18:00

Comment            = phase sensitive noesy
Data_Format        = 2D COMPLEX COMPLEX
Dim_Size           = 512, 819
Dim_Title          = Proton Proton
Dim_Units          = [ppm] [ppm]
Dimensions         = X Y
Site               = JNM-ECA800
Spectrometer       = DELTA2_NMR

Field_Strength     = 18.79305132[T] (800[MHz])
X_Acq_Duration     = 73.07264[ms]
X_Domain           = 1H
X_Freq             = 800.14000039[MHz]
X_Offset           = 5[ppm]
X_Points           = 1024
X_Prescans         = 4
X_Resolution       = 13.68501261[Hz]
X_Sweep            = 14.01345291[kHz]
X_Sweep_Clipped    = 11.21076233[kHz]
Y_Domain           = 1H
Y_Freq             = 800.14000039[MHz]
Y_Offset           = 5[ppm]
Y_Points           = 256
Y_Prescans         = 0
Y_Resolution       = 43.76260363[Hz]
Y_Sweep            = 11.20322653[kHz]
Irr_Domain         = Proton
Irr_Freq           = 800.14000039[MHz]
Irr_Offset         = 5[ppm]
Tri_Domain         = Proton
Tri_Freq           = 800.14000039[MHz]
Tri_Offset         = 5[ppm]
Clipped            = FALSE
Scans              = 16
Total_Scans        = 4096

Relaxation_Delay   = 2[s]
Recvr_Gain         = 44
Temp_Get           = 22.4[dC]
Mix_Time           = 0.7[s]
X_Acq_Time         = 73.07264[ms]
X_Atn              = 3.2[dB]
X_Pulse            = 15.5[us]
Y_Acq_Time         = 22.85056[ms]
Y_P0_Correction    = 0
Y_P1_Correction    = 180.0
Irr_Mode           = Off
Tri_Mode           = Off
Dante_Presat       = FALSE
Initial_Wait       = 1[s]
Phase_1            = {(0, 180, 0, 180, 0, 180,
Repetition_Time    = 2.07307264[s]
Scramble           = 1[ms]
T1                 = 24.79[us]
T1_Cal1            = 24.79[us]
T1_Cal2            = 69.42[us]
T1_Cal3            = 0.11405[ms]
T1_Cal4            = 0.15868[ms]
T1_Cal5            = 0.20331[ms]
T1_Cal6            = 0.24794[ms]
Zf_Factor          = 3
```

galangalditerpene C (3)

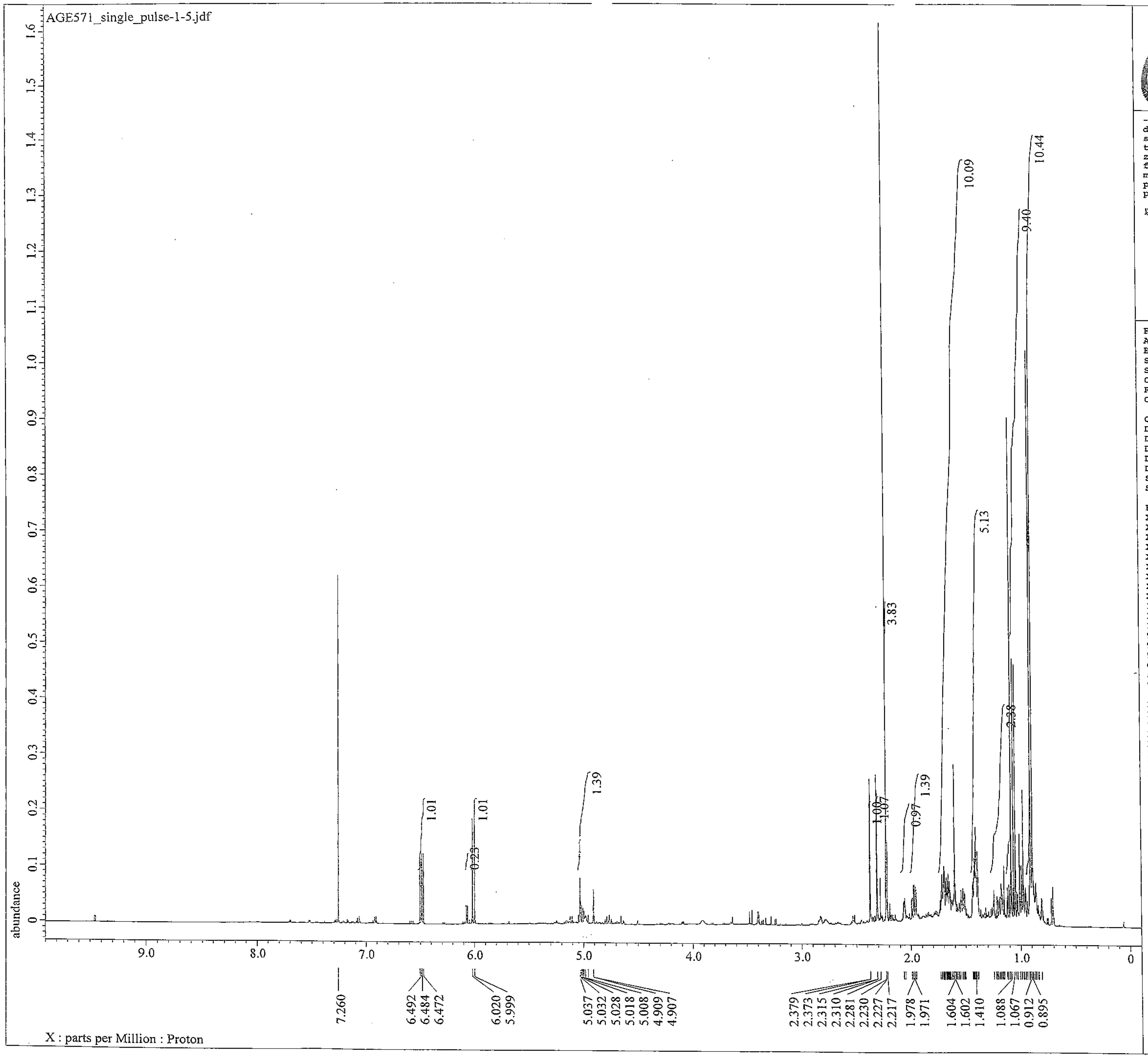

---- PROCESSING PARAMETERS ----
dc_balance(0, FALSE)
sexp(0.2[Hz], 0.0[s])
trapezoid(0[%], 0[%], 80[%], 100[%])
zerofill(1)
fft(1, TRUE, TRUE)
machinephase
ppm
phase(-6, 0, 68.61601[%])

以下に由来:: AGE571_single_pulse-1-1.jdf

Filename = C:\Users\delta\Documents\J
Author = delta
Experiment = single_pulse.jxp
Sample_Id = AGE571
Solvent = CHLOROFORM-D
Creation_Time = 15-NOV-2012 10:45:39
Revision_Time = 15-NOV-2012 11:02:40
Current_Time = 15-NOV-2012 11:03:06

Comment = single_pulse
Data_Format = 1D COMPLEX
Dim_Size = 52429
Dim_Title = Proton
Dim_Units = [ppm]
Dimensions = X
Site = JNM-ECA800
Spectrometer = DELTA2_NMR

Field_Strength = 18.79305132[T] (800[MHz])
X_Acq_Duration = 4.36207616[s]
X_Domain = 1H
X_Freq = 800.14000039[MHz]
X_Offset = 5[ppm]
X_Points = 65536
X_Prescans = 1
X_Resolution = 0.22924863[Hz]
X_Sweep = 15.02403846[kHz]
X_Sweep_Clipped = 12.01923077[kHz]
Irr_Domain = Proton
Irr_Freq = 800.14000039[MHz]
Irr_Offset = 5[ppm]
Tri_Domain = Proton
Tri_Freq = 800.14000039[MHz]
Tri_Offset = 5[ppm]
Clipped = FALSE
Scans = 32
Total_Scans = 32

Relaxation_Delay = 5[s]
Recvr_Gain = 36
Temp_Get = 20.9[dC]
X_90_Width = 16[us]
X_Acq_Time = 4.36207616[s]
X_Angle = 45[deg]
X_Atn = 3[dB]
X_Pulse = 8[us]
Irr_Mode = Off
Tri_Mode = Off
Dante_Presat = FALSE
Initial_Wait = 1[s]
Repetition_Time = 9.36207616[s]

galangalditerpene C (3)

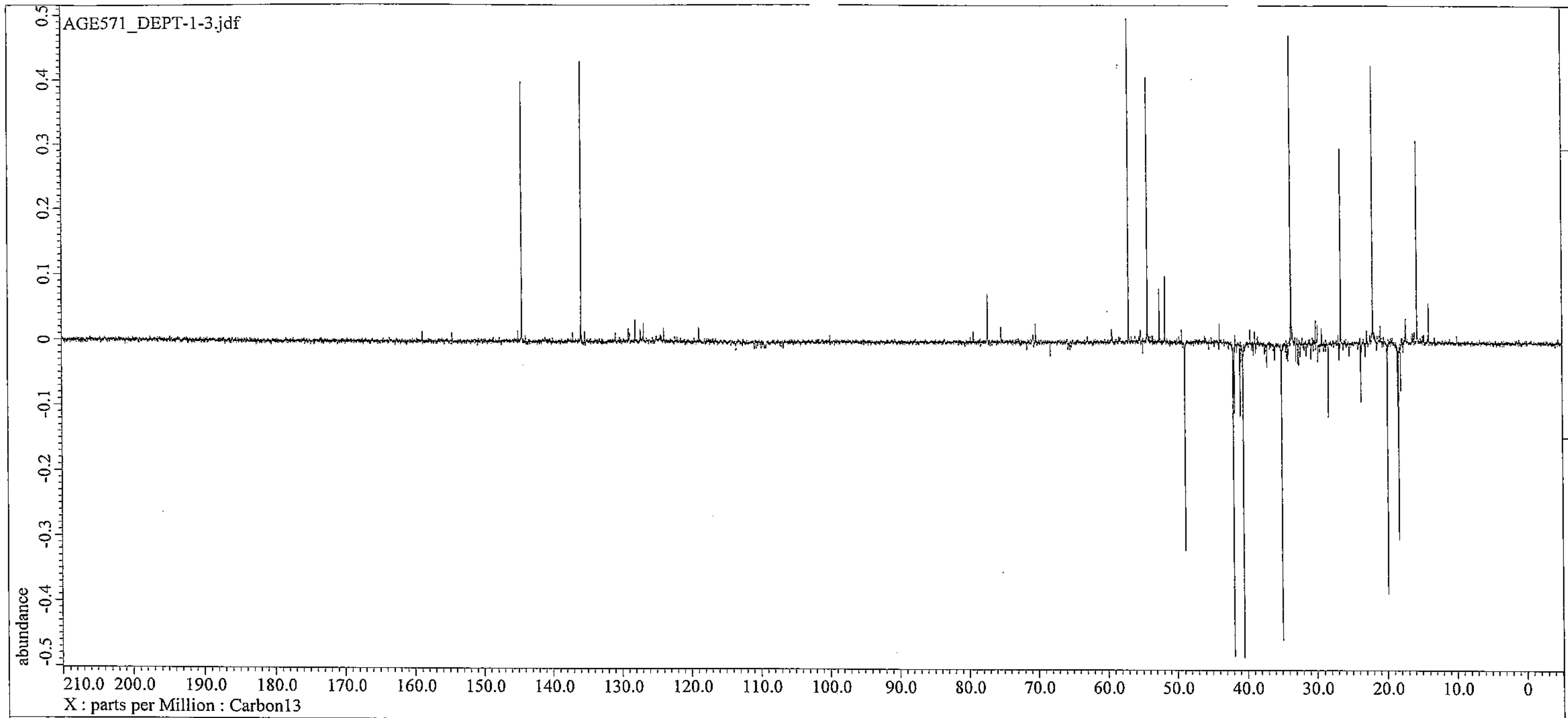

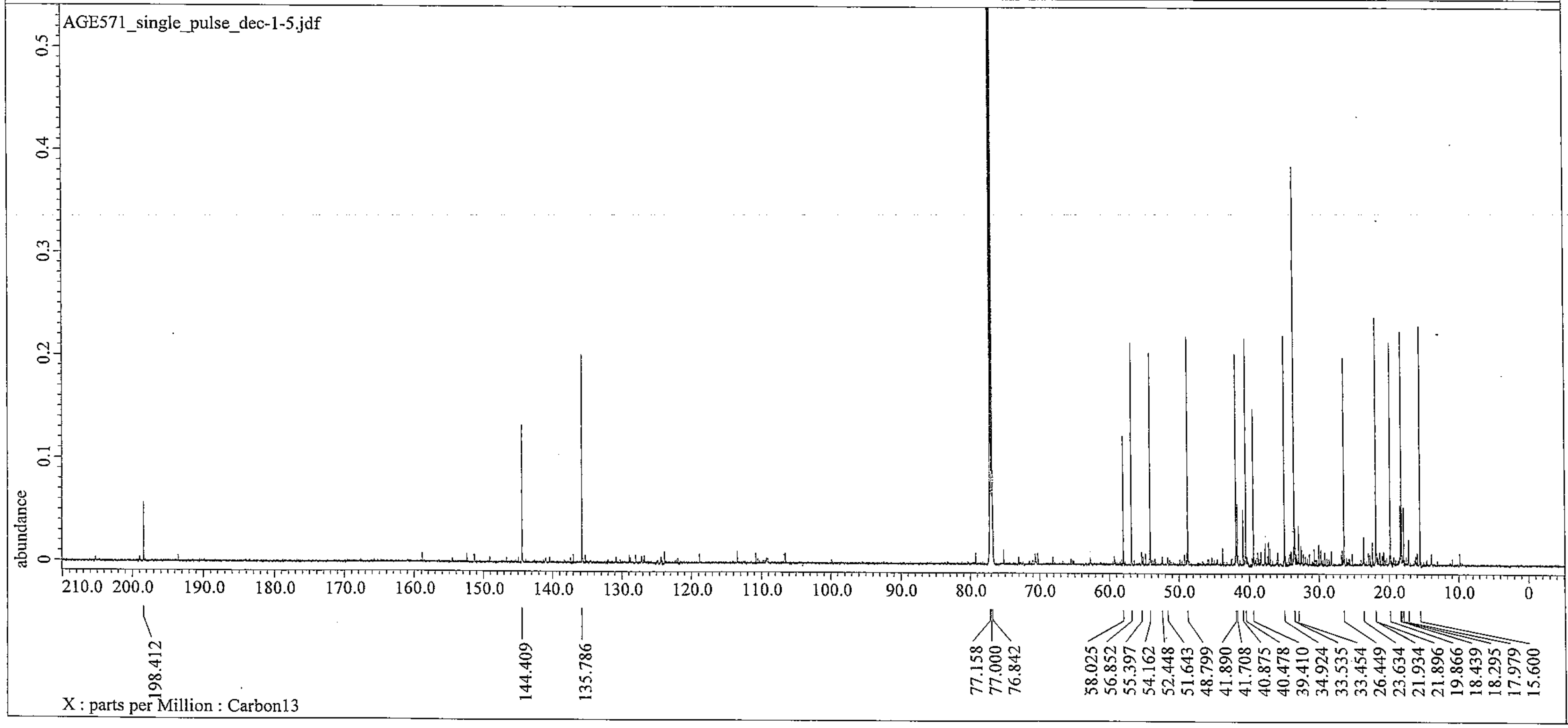

---- PROCESSING PARAMETERS ----
dc_balance(0, FALSE)
sexp(2.0[Hz], 0.0[s])
trapezoid(0[%], 0[%], 80[%], 100[%])
zerofill(1)
fft(1, TRUE, TRUE)
machinephase
ppm
phase(0.75, 0, 58.98566[%])
base_correct(None, 10, Smooth)

Filename	= C:\Users\delta\Documents\J
Author	= delta
Experiment	= single_pulse_dec.jxp
Sample_Id	= AGE571
Solvent	= CHLOROFORM-D
Creation_Time	= 15-NOV-2012 10:51:21
Revision_Time	= 16-NOV-2012 10:39:18
Current_Time	= 16-NOV-2012 10:41:49
Comment	= single pulse decoupled gat
Data_Format	= 1D REAL
Dim_Size	= 52429
Dim_Title	= Carbon13
Dim_Units	= [ppm]
Dimensions	= X
Site	= JNM-ECA800
Spectrometer	= DELTA2_NMR
Field_Strength	= 18.79305132[T] (800[MHz])
X_Acq_Duration	= 1.03809024[s]
X_Domain	= 13C
X_Freq	= 201.19534229[MHz]
X_Offset	= 100[ppm]
X_Points	= 65536
X_Prescans	= 4
X_Resolution	= 0.96330739[Hz]
X_Sweep	= 63.13131313[kHz]
X_Sweep_Clipped	= 50.50505051[kHz]
Irr_Domain	= Proton
Irr_Freq	= 800.14000039[MHz]
Irr_Offset	= 5[ppm]
Clipped	= TRUE
Scans	= 10000
Total_Scans	= 10000
Relaxation_Delay	= 2[s]
Recvr_Gain	= 60
Temp_Get	= 21.1[dC]
X_90_Width	= 11.2[us]
X_Acq_Time	= 1.03809024[s]
X_Angle	= 30[deg]
X_Atn	= 2.6[dB]
X_Pulse	= 3.73333333[us]
Irr_Atn_Dec	= 14.35[dB]
Irr_Atn_Noe	= 14.35[dB]
Irr_Noise	= WALTZ
Irr_Pwidth	= 57[us]
Decoupling	= TRUE
Initial_Wait	= 1[s]
Noe	= TRUE
Noe_Time	= 2[s]
Repetition_Time	= 3.03809024[s]

galangalditerpene C (3)

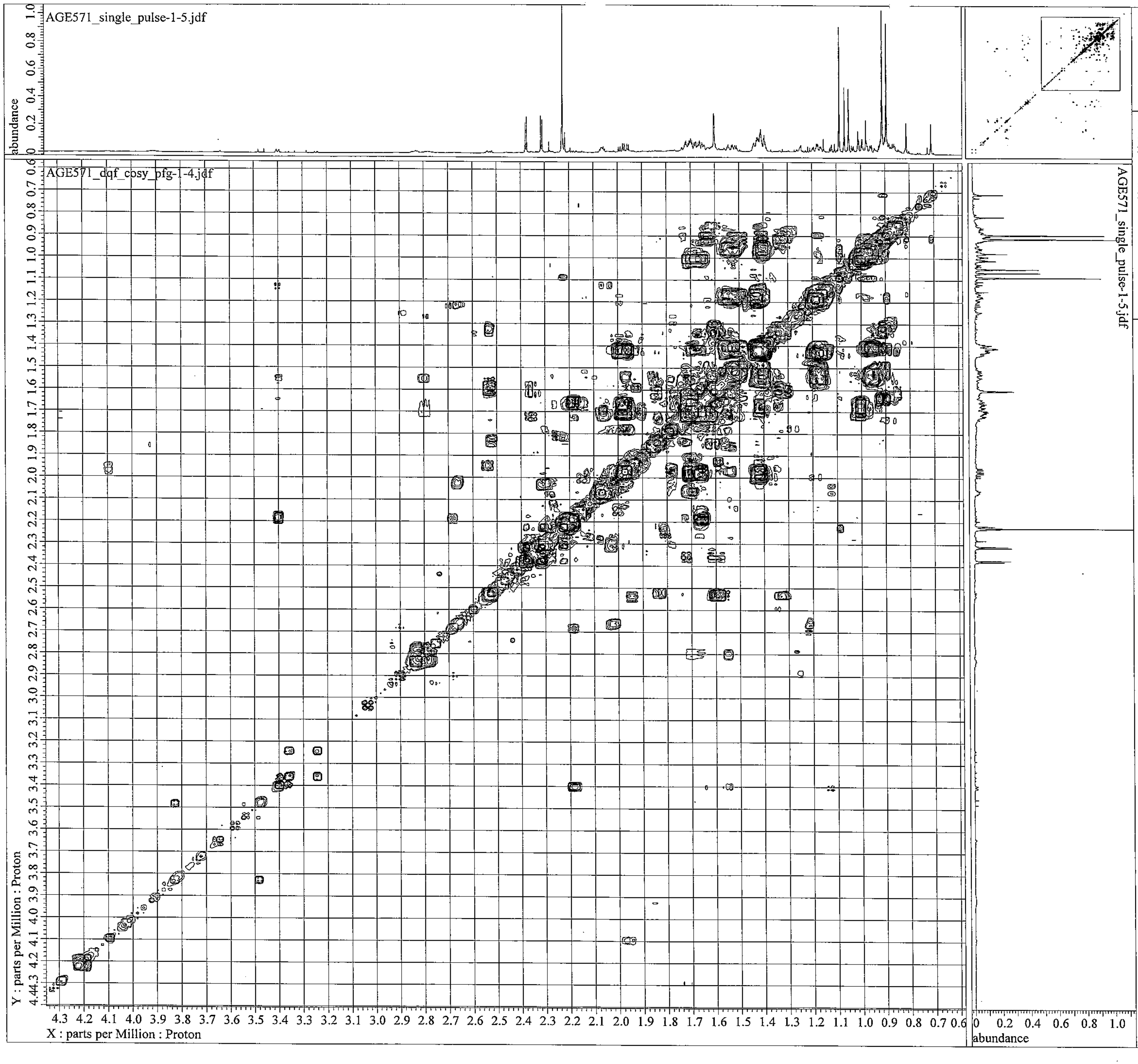


```
---- PROCESSING PARAMETERS ----
sinbell_auto
fft( 1, TRUE, TRUE )
ppm
[transpose]
sinbell_auto
zerofill( 4 )
fft( 1, TRUE, TRUE )
ppm
abs
symmetrize( Cosy, 24 )
[transpose]
```

```
Filename          = C:\Users\delta\Documents\J
Author            = delta
Experiment        = dqf_cosy_pfg.jxp
Sample_Id         = AGE571
Solvent           = CHLOROFORM-D
Creation_Time     = 15-NOV-2012 21:50:15
Revision_Time     = 16-NOV-2012 10:43:31
Current_Time      = 16-NOV-2012 10:47:30

Comment           = gradient absolute value DQ
Data_Format       = 2D REAL REAL
Dim_Size          = 1024, 1024
Dim_Title         = Proton Proton
Dim_Units         = [ppm] [ppm]
Dimensions        = X Y
Site              = JNM-ECA800
Spectrometer      = DELTA2_NMR

Field_Strength    = 18.79305132[T] (800[MHz])
X_Acq_Duration    = 0.1599488[s]
X_Domain          = 1H
X_Freq            = 800.14000039[MHz]
X_Offset          = 4[ppm]
X_Points          = 1280
X_Prescans        = 4
X_Resolution      = 6.25200064[Hz]
X_Sweep           = 8.00256082[kHz]
X_Sweep_Clipped   = 6.40204866[kHz]
Y_Domain          = 1H
Y_Freq            = 800.14000039[MHz]
Y_Offset          = 4[ppm]
Y_Points          = 256
Y_Prescans        = 0
Y_Resolution      = 25.00480092[Hz]
Y_Sweep           = 6.40122904[kHz]
Irr_Domain        = Proton
Irr_Freq          = 800.14000039[MHz]
Irr_Offset        = 5[ppm]
Tri_Domain        = Proton
Tri_Freq          = 800.14000039[MHz]
Tri_Offset        = 5[ppm]
Clipped           = FALSE
Scans             = 8
Total_Scans       = 2048

Relaxation_Delay = 1.5[s]
Recvr_Gain        = 34
Temp_Get          = 20.8[dC]
X_Acq_Time        = 0.1599488[s]
X_Atn             = 3[dB]
X_Pulse           = 16[us]
Y_Acq_Time        = 39.99232[ms]
Irr_Mode          = Off
Tri_Mode          = Off
Dante_Presat      = FALSE
Grad_1            = 1[ms]
Grad_1_Amp        = 60[mT/m]
Grad_2            = 1[ms]
Grad_2_Amp        = 45[mT/m]
Grad_3            = 1[ms]
Grad_3_Amp        = 0.15[T/m]
Grad_Recover      = 0.1[ms]
Grad_Selection    = 4:3:10
Grad_Shape        = SINE
Initial_Wait      = 1[s]
Repetition_Time   = 1.6599488[s]
Scramble          = 1[ms]
T1                = 1[us]
Zf_Factor         = 4
```

galangalditerpene C (3)

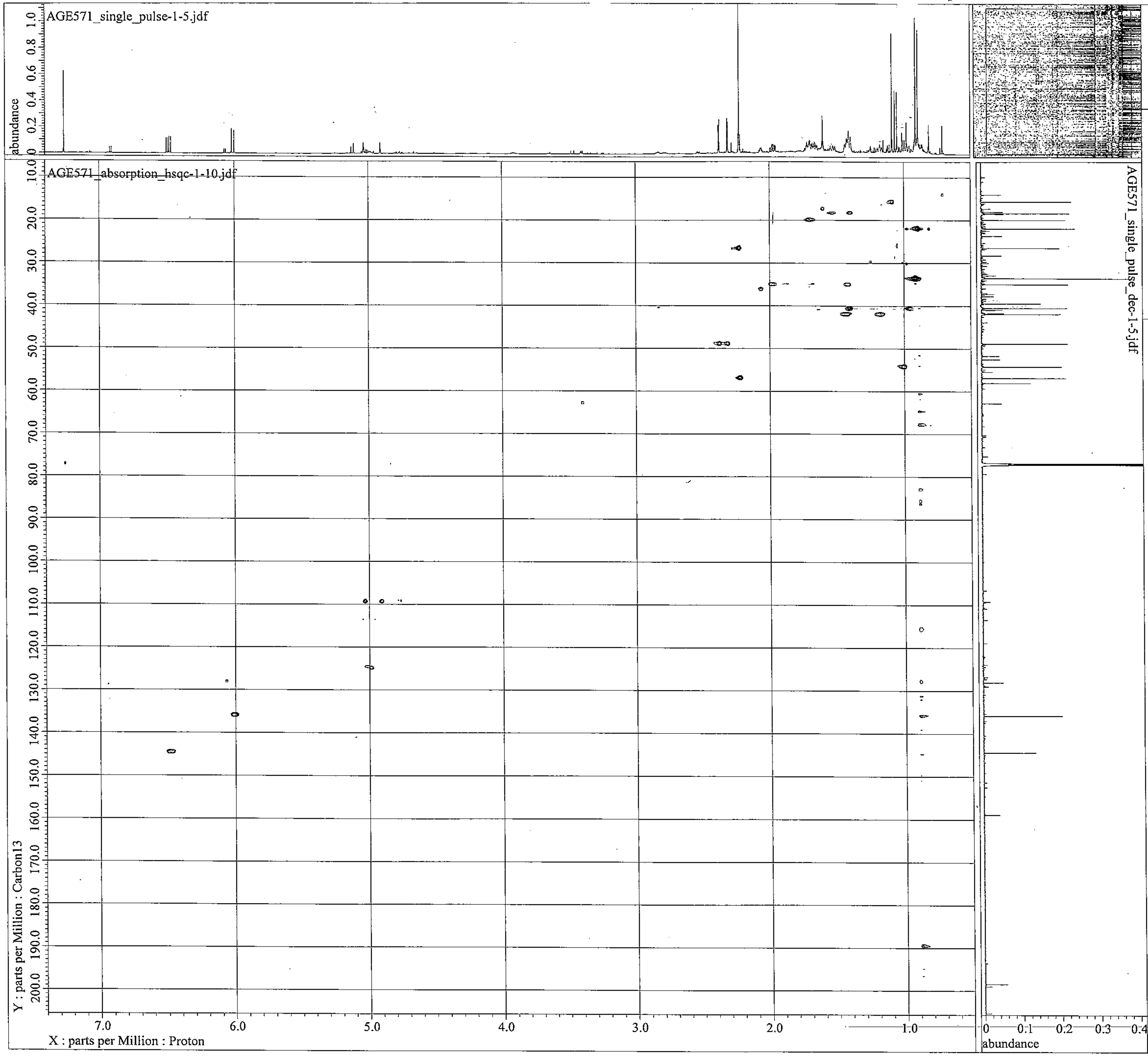


```
---- PROCESSING PARAMETERS ----
sexp( 10.0[Hz], 0.0[s] )
trapezoid( 0[%], 0[%], 80[%], 100[%] )
zerofill( 1 )
fft( 1, TRUE, TRUE )
ppm
machinephase
phase( 3.16575, -60.417, 88.75306[%] )
[transpose]
sexp( 50.0[Hz], 0.0[s] )
trapezoid( 0[%], 0[%], 80[%], 100[%] )
zerofill( 2 )
fft( 1, TRUE, TRUE )
ppm
machinephase
[transpose]

Filename          = C:\Users\delta\Documents\J
Author            = delta
Experiment        = hsqc_dec_phase_pfgzz.jxp
Sample_Id         = AGE571
Solvent           = CHLOROFORM-D
Creation_Time     = 15-NOV-2012 22:48:26
Revision_Time     = 16-NOV-2012 10:54:52
Current_Time      = 16-NOV-2012 10:56:20

Comment           = absorption_hsqc
Data_Format       = 2D COMPLEX COMPLEX
Dim_Size          = 819, 512
Dim_Title         = Proton Carbon13
Dim_Units         = [ppm] [ppm]
Dimensions        = X Y
Site              = JNM-ECA800
Spectrometer      = DELTA2_NMR

Field_Strength    = 18.79305132[T] (800[MHz])
X_Acq_Duration    = 0.12787712[s]
X_Domain          = 1H
X_Freq            = 800.14000039[MHz]
X_Offset          = 4[ppm]
X_Points          = 1024
X_Prescans        = 4
X_Resolution      = 7.82000721[Hz]
X_Sweep           = 8.00768738[kHz]
X_Sweep_Clipped   = 6.4061499[kHz]
Y_Domain          = 13C
Y_Freq            = 201.19534229[MHz]
Y_Offset          = 105.0[ppm]
Y_Points          = 256
Y_Prescans        = 0
Y_Resolution      = 165.23900169[Hz]
Y_Sweep           = 42.30118443[kHz]
Tri_Domain        = Proton
Tri_Freq          = 800.14000039[MHz]
Tri_Offset        = 5[ppm]
Clipped           = FALSE
Scans             = 8
Total_Scans       = 2048

Relaxation_Delay  = 1.5[s]
Recvr_Gain        = 34
Temp_Get          = 20.8[dC]
X_Acq_Time        = 0.12787712[s]
X_Atn             = 3[dB]
X_Pulse           = 16[us]
Y_Acq_Time        = 6.05184[ms]
Y_Atn             = 2.6[dB]
Y_P1_Correction   = 720
Y_Pulse           = 11.2[us]
Irr_Atn_Dec       = 20.985[dB]
Irr_Noise         = MPF10
Irr_Pwidth        = 93[us]
Tri_Mode          = Off
Composite_Pulse   = FALSE
Dante_Presat      = FALSE
Decoupling        = TRUE
Grad_1_Amp        = 0.105[T/m]
Grad_2_Amp        = 30[mT/m]
Initial_Wait      = 1[s]
J_Constant        = 140[Hz]
Phase_Qd          = {(0, 180}, {90, 270}}
Purge             = 0[ms]
Repetition_Time   = 1.62787712[s]
T1                = 0.472[us]
T1_Cal1           = -17.258[us]
T1_Cal2           = -11.348[us]
T1_Cal3           = -5.438[us]
T1_Cal4           = 0.472[us]
T1_Cal5           = 6.382[us]
```

galangalditerpene C (3)

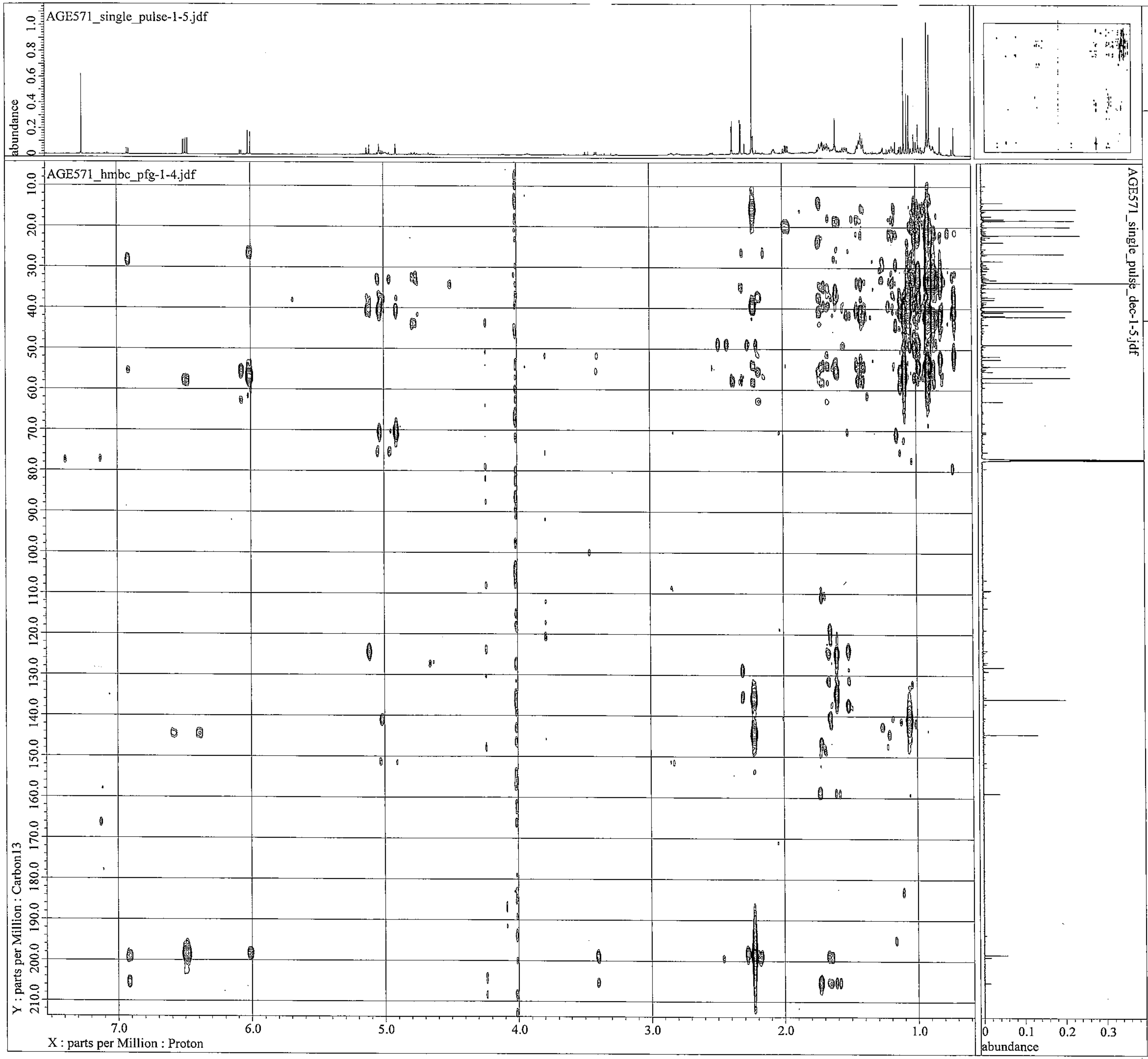


```
---- PROCESSING PARAMETERS ----
gauss( 5.0[Hz], 0.0[s] )
sinbell_auto
zerofill( 1 )
fft( 1, TRUE, TRUE )
ppm
[transpose]
sinbell4( -60, 160 )
trapezoid( 0[%], 5[%], 80[%], 100[%] )
zerofill( 2 )
fft( 1, TRUE, TRUE )
ppm
abs
[transpose]
```

```
Filename          = C:\Users\delta\Documents\J
Author            = delta
Experiment        = hmbc_pfg.jxp
Sample_Id         = AGE571
Solvent           = CHLOROFORM-D
Creation_Time     = 16-NOV-2012 00:41:09
Revision_Time     = 16-NOV-2012 10:58:53
Current_Time      = 16-NOV-2012 11:00:04

Comment           = gradient enhanced HMBC
Data_Format       = 2D REAL REAL
Dim_Size          = 512, 1638
Dim_Title         = Carbon13 Proton
Dim_Units         = [ppm] [ppm]
Dimensions        = X Y
Site              = JNM-ECA800
Spectrometer      = DELTA2_NMR

Field_Strength    = 18.79305132[T] (800[MHz])
X_Acq_Duration    = 0.25575424[s]
X_Domain          = 1H
X_Freq            = 800.14000039[MHz]
X_Offset          = 4[ppm]
X_Points          = 2048
X_Prescans        = 4
X_Resolution      = 3.9100036[Hz]
X_Sweep           = 8.00768738[kHz]
X_Sweep_Clipped   = 6.4061499[kHz]
Y_Domain          = 13C
Y_Freq            = 201.19534229[MHz]
Y_Offset          = 100[ppm]
Y_Points          = 256
Y_Prescans        = 0
Y_Resolution      = 196.49144869[Hz]
Y_Sweep           = 50.30181087[kHz]
Tri_Domain        = Proton
Tri_Freq          = 800.14000039[MHz]
Tri_Offset        = 5[ppm]
Clipped           = FALSE
Scans             = 32
Total_Scans       = 8192

Relaxation_Delay = 1.5[s]
Recvr_Gain        = 34
Temp_Get          = 20.7[dC]
X_Acq_Time        = 0.25575424[s]
X_Atn             = 3[dB]
X_Pulse           = 16[us]
Y_Acq_Time        = 5.08928[ms]
Y_Atn             = 2.6[dB]
Y_Pulse           = 11.2[us]
Tri_Mode          = Off
Dante_Presat      = FALSE
Delta             = 62.5[ms]
Delta_1           = 3.83288616[ms]
Delta_2           = 3.43642612[ms]
Delta_3           = 3.11429461[ms]
Grad_1            = 1[ms]
Grad_1_Amp        = 0.18[T/m]
Grad_2            = 1[ms]
Grad_2_Amp        = 0.18[T/m]
Grad_3            = 1[ms]
Grad_3_Amp        = 90.54325956[mT/m]
Grad_Lpf          = 1[ms]
Grad_Lpf1_Amp     = 45[mT/m]
Grad_Lpf2_Amp     = -25.71428571[mT/m]
Grad_Lpf3_Amp     = -12.85714286[mT/m]
Grad_Lpf4_Amp     = -6.42857143[mT/m]
Grad_Recover      = 0.1[ms]
Grad_Selection    = 13C = 1.988:1.988:1
Grad_Shape        = SINE
Initial_Wait      = 1[s]
```

galangalditerpene C (3)

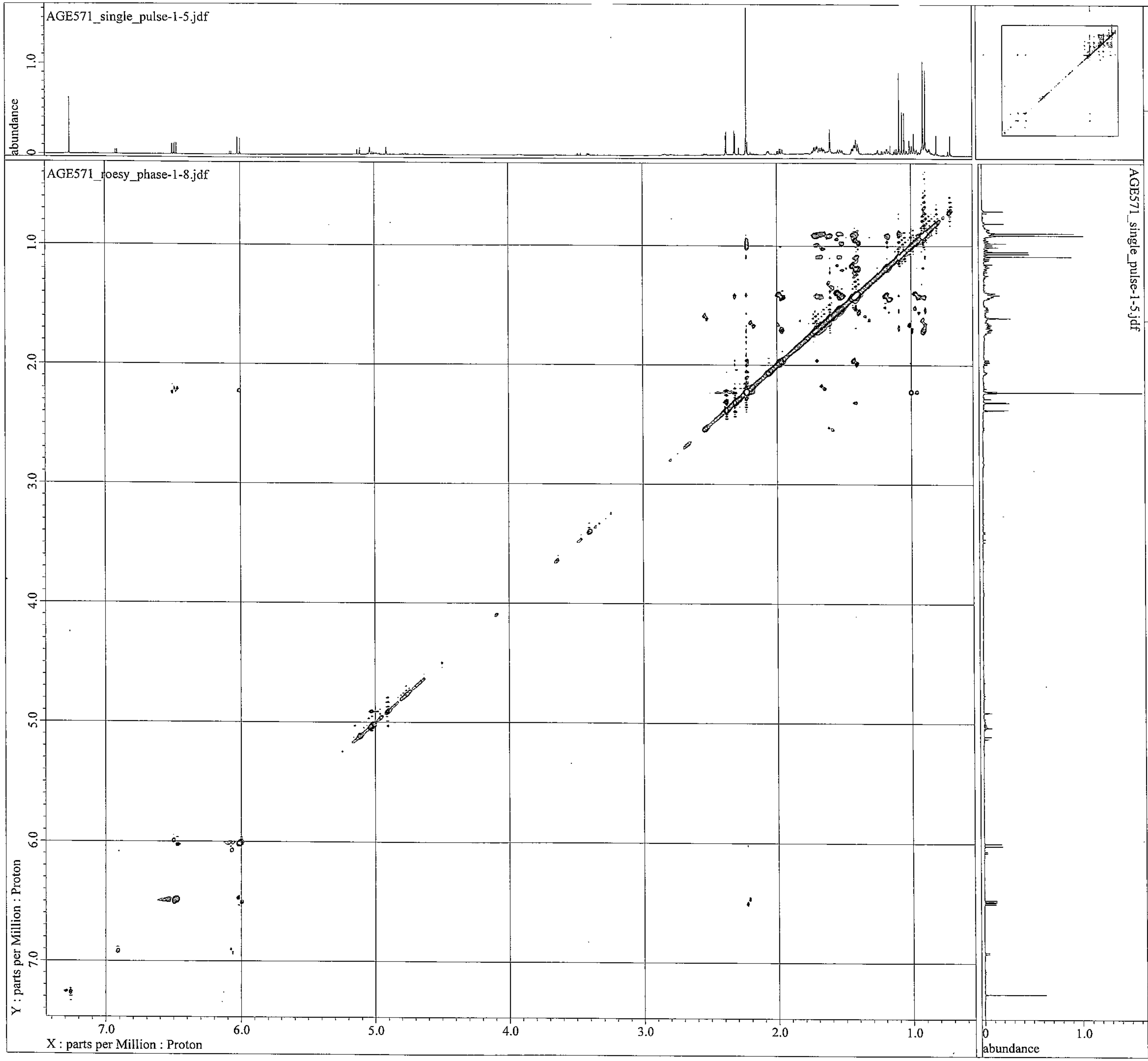


```
---- PROCESSING PARAMETERS ----
sexp( 5.0[Hz], 0.0[s] )
trapezoid( 0[%], 0[%], 80[%], 100[%] )
zerofill( 1 )
fft( 1, TRUE, TRUE )
ppm
machinephase
phase( -8.5, -30, 67.97066[%] )
[transpose]
sexp( 5.0[Hz], 0.0[s] )
trapezoid( 0[%], 0[%], 80[%], 100[%] )
zerofill( 2 )
fft( 1, TRUE, TRUE )
ppm
machinephase
[transpose]
```

```
Filename           = C:\Users\delta\Documents\
Author             = delta
Experiment         = roesy_phase.jxp
Sample_Id          = AGE571
Solvent            = CHLOROFORM-D
Creation_Time      = 16-NOV-2012 04:50:51
Revision_Time      = 16-NOV-2012 11:13:32
Current_Time       = 16-NOV-2012 11:14:55

Comment            = absorption roesy
Data_Format        = 2D COMPLEX COMPLEX
Dim_Size           = 512, 819
Dim_Title          = Proton Proton
Dim_Units          = [ppm] [ppm]
Dimensions         = X Y
Site               = JNM-ECA800
Spectrometer       = DELTA2_NMR

Field_Strength     = 18.79305132[T] (800[MHz])
X_Acq_Duration     = 0.10231808[s]
X_Domain           = 1H
X_Freq             = 800.14000039[MHz]
X_Offset           = 4[ppm]
X_Points           = 1024
X_Prescans         = 4
X_Resolution       = 9.77344376[Hz]
X_Sweep            = 10.00800641[kHz]
X_Sweep_Clipped    = 8.00640512[kHz]
Y_Domain           = 1H
Y_Freq             = 800.14000039[MHz]
Y_Offset           = 4[ppm]
Y_Points           = 256
Y_Prescans         = 0
Y_Resolution       = 31.2600032[Hz]
Y_Sweep            = 8.00256082[kHz]
Irr_Domain         = Proton
Irr_Freq           = 800.14000039[MHz]
Irr_Offset         = 5[ppm]
Tri_Domain         = Proton
Tri_Freq           = 800.14000039[MHz]
Tri_Offset         = 5[ppm]
Clipped            = FALSE
Scans              = 16
Total_Scans        = 4096

Relaxation_Delay   = 1.5[s]
Recvr_Gain         = 34
Temp_Get           = 20.6[dC]
Mix_Time           = 0.5[s]
X_Acq_Time         = 0.10231808[s]
X_Atn              = 3[dB]
X_Pulse            = 16[us]
X_Spinlock_Atn     = 21[dB]
X_Spinlock_Mode    = 18[dB]down
Y_Acq_Time         = 31.98976[ms]
Y_P1_Correction    = 180
Irr_Mode           = Off
Tri_Mode           = Off
Dante_Presat       = FALSE
Initial_Wait       = 1[s]
Phase_1            = {(0, 180, 180, 0, 90, 270
Repetition_Time    = 1.60231808[s]
Spinlock_Strength  = 1.953125[kHz]
T1                 = 52.24[us]
T1_Cal1            = 52.24[us]
T1_Cal2            = 0.11472[ms]
T1_Cal3            = 0.1772[ms]
T1_Cal4            = 0.23968[ms]
T1_Cal5            = 0.30216[ms]
T1_Cal6            = 0.36464[ms]
Temp1              = 18[dB]
Temp2              = 8
```
