# Supplementary material for: Labdane-Type Diterpenes, Galangalditerpenes A–C, with Melanogenesis Inhibitory Activity from the Fruit of Alpinia galanga
Source: Molecules. 2017 Dec 20;22(12):2279. doi: 10.3390/molecules22122279 (PMC6149739; doi:10.3390/molecules22122279)
Supplement: Supplementary file 1 [file molecules-22-02279-s001.zip › supplementary.v2.pdf]

Supplementary Materials:

Labdane-type Diterpenes, Galangalditerpenes A–C, with Melanogenesis Inhibitory Activity from the Fruit of *Alpinia galanga*

Yoshiaki Manse [1], Kiyofumi Ninomiya [1,2], Ryosuke Nishi [1], Yoshinori Hashimoto [1], Saowanee Chaipech [1,3], Osamu Muraoka [1,2] and Toshio Morikawa [1,2,*]

[1] Pharmaceutical Research and Technology Institute, Kindai University, 3-4-1 Kowakae, Higashi-osaka, Osaka 577-8502, Japan; yoshiaki4849sa735@gmail.com (Y.M.); ninomiya@phar.kindai.ac.jp (K.N.); kojirou922@gmail.com (R.N.); yoshinorihashimoto0608@gmail.com (Y.H.); chaipechann@hotmail.com (S.C.); muraoka@phar.kindai.ac.jp (O.M.)

[2] Antiaging Center, Kindai University, 3-4-1 Kowakae, Higashi-osaka, Osaka 577-8502, Japan

[3] Faculty of Agro-Industry, Rajamangala University of Technology Srivijaya, Thungyai, Nakhon Si Thammarat 80240, Thailand

* Correspondence: morikawa@kindai.ac.jp; Tel.: +81-6-4307-4306

Table S1. Effects on activity of tyrosinase from mushroom.

**Treatment**	**Inhibition (%)**					
	**Substrate: L-Tyrosine**			**Substrate: L-DOPA**		
	**0 μM**	**10 μM**	**100 μM**	**0 μM**	**10 μM**	**100 μM**
Galangalditerpene A (**1**)	0.0 ± 2.7	5.2 ± 2.1	27.8 ± 2.1 **	0.0 ± 3.3	4.6 ± 1.0	9.2 ± 1.2 *
Galangalditerpene B (**2**)	0.0 ± 2.5	−0.2 ± 0.6	2.6 ± 0.9	0.0 ± 1.7	3.9 ± 1.7	0.7 ± 0.8
Galangalditerpene C (**3**)	0.0 ± 0.8	−6.3 ± 1.8	1.4 ± 6.7	0.0 ± 2.5	−5.0 ± 0.4	−2.3 ± 1.0
Clovane-2*β*,9*α*-diol (**4**)	0.0 ± 1.6	1.2 ± 3.7	8.2 ± 1.6	0.0 ± 1.3	0.8 ± 1.5	−5.8 ± 1.2 *
Caryolane-1,9*β*-diol (**5**)	0.0 ± 1.4	5.4 ± 1.0 *	3.6 ± 1.6	0.0 ± 2.9	10.7 ± 3.0 *	14.7 ± 1.5 **
(−)-2-Oxoisodauc-5-en-12-al (**6**)	0.0 ± 1.1	−2.4 ± 1.1	−1.0 ± 1.4	0.0 ± 3.9	1.8 ± 2.0	0.7 ± 1.8
Kobusone (**7**)	0.0 ± 0.5	1.2± 1.7	0.7 ± 1.5	0.0 ± 1.2	−10.1 ± 2.2 *	−12.5 ± 2.5 **
Galanolactone (**8**)	0.0 ± 1.3	2.3 ± 1.5	6.6 ± 1.4 *	0.0 ± 1.9	−6.0 ± 1.1 *	−5.9 ± 1.0 *
(*E*)-15,16-Bisnorlabda-8(17),11-diene-13-one (**9**)	0.0 ± 1.0	2.1 ± 0.9	2.2 ± 1.1	0.0 ± 2.8	−9.7 ± 1.5 *	−4.8 ± 1.6
**Substrate: L-Tyrosine**	**Inhibition (%)**					
**Treatment**	**0 μM**	**10 μM**	**30 μM**	**100 μM**	**300 μM**	**IC_{50} (μM)**
Kojic acid [13,24,26-29]	0.0 ± 2.4	12.2 ± 3.3	46.4 ± 2.6 **	66.5 ± 2.1 **	96.8 ± 0.9 **	43.6
**Substrate: L-DOPA**	**Inhibition (%)**					
**Treatment**	**0 μM**	**10 μM**	**30 μM**	**100 μM**	**300 μM**	**IC_{50} (μM)**
Kojic acid [13,24,26-29]	0.0 ± 0.9	22.3 ± 2.1 **	50.6 ± 0.6 **	78.2 ± 0.7 **	89.3 ± 0.3 **	29.6

Each value represents the mean ± S.E.M. (n = 4); asterisks denote significant differences from the control group, * $p < 0.05$, ** $p < 0.01$; commercial kojic acid was purchased from Nakalai Tesque Inc., (Kyoto, Japan).
